# Investigating the stability of aromatic carboxylic acids in hydrated magnesium sulfate under UV irradiation to assist detection of organics on Mars

**DOI:** 10.1038/s41598-024-66669-8

**Published:** 2024-07-10

**Authors:** Andrew Alberini, Teresa Fornaro, Cristina García-Florentino, Malgorzata Biczysko, Iratxe Poblacion, Julene Aramendia, Juan Manuel Madariaga, Giovanni Poggiali, Álvaro Vicente-Retortillo, Kathleen C. Benison, Sandra Siljeström, Sole Biancalani, Christian Lorenz, Edward A. Cloutis, Dan M. Applin, Felipe Gómez, Andrew Steele, Roger C. Wiens, Kevin P. Hand, John R. Brucato

**Affiliations:** 1 INAF- Astrophysical Observatory of Arcetri, L.go E. Fermi 5, 50125 Firenze, Italy; 2 Department of Physics and Astronomy, University of Florence, Via Giovanni Sansone 1, Sesto Fiorentino, 50019 Florence, Italy; 3 Department of Analytical Chemistry, University of the Basque Country UPV/EHU, 48080 Bilbao, Spain; 4 College of Science, Shanghai University, 99 Shangda Road, Shanghai, 200444 China; 5 LESIA - Observatoire de Paris, CNRS, Université Paris Cité, Université PSL, Sorbonne Université, 5 Place Jules Janssen, 92190 Meudon, France; 6 Centro de Astrobiología (CAB), CSIC-INTA, Torrejón de Ardoz, Spain; 7 Department of Geology and Geography, West Virginia University, Morgantown, WV USA; 8 RISE Research Institutes of Sweden, Stockholm, Sweden; 9 Department of Physics, University of Trento, Via Sommarive 14, 38123 Povo, Italy; 10 Italian Space Angency (ASI), Viale del Politecnico Snc, 00133 Rome, Italy; 11 Department of Earth Sciences, University of Florence, Via G. La Pira 4, 50121 Florence, Italy; 12 Department of Biology, University of Naples Federico II, Via Cinthia, 80126 Naples, Italy; 13 Centre for Terrestrial and Planetary Exploration, University of Winnipeg, Winnipeg, MB R3B 2E9 Canada; 14 Carnegie Institute for Science, Washington, DC USA; 15 Earth, Atmospheric, and Planetary Sciences, Purdue University, West Lafayette, IN USA; 16 Jet Propulsion Laboratory, California Institute of Technology, Pasadena, CA USA

**Keywords:** Aromatic organic compounds, FTIR spectroscopy, UV irradiation, Signs of biological activity, Mars 2020 Perseverance mission, Astrobiology, Astrobiology

## Abstract

The Scanning Habitable Environments with Raman and Luminescence for Organics and Chemicals (SHERLOC) instrument onboard the Mars 2020 Perseverance rover detected so far some of the most intense fluorescence signals in association with sulfates analyzing abraded patches of rocks at Jezero crater, Mars. To assess the plausibility of an organic origin of these signals, it is key to understand if organics can survive exposure to ambient Martian UV after exposure by the Perseverance abrasion tool and prior to analysis by SHERLOC. In this work, we investigated the stability of organo-sulfate assemblages under Martian-like UV irradiation and we observed that the spectroscopic features of phthalic and mellitic acid embedded into hydrated magnesium sulfate do not change for UV exposures corresponding to at least 48 Martian sols and, thus, should still be detectable in fluorescence when the SHERLOC analysis takes place, thanks to the photoprotective properties of magnesium sulfate. In addition, different photoproduct bands diagnostic of the parent carboxylic acid molecules could be observed. The photoprotective behavior of hydrated magnesium sulfate corroborates the hypothesis that sulfates might have played a key role in the preservation of organics on Mars, and that the fluorescence signals detected by SHERLOC in association with sulfates could potentially arise from organic compounds.

## Introduction

The detection of organic compounds in the Martian rocks is important for the NASA Mars 2020 Perseverance rover to assess the astrobiology relevance of the Martian samples under investigation at Jezero crater and collected for future return to Earth. In the current Martian environment, organic compounds undergo degradation primarily through oxidation and photolysis/radiolysis^1–3^. Indeed, the current atmosphere is very thin, with pressure of about $6$ mbar at the surface, and is composed almost entirely of carbon dioxide (i.e. 96%). The photon flux with wavelengths less than 190 nm is absorbed by atmospheric carbon dioxide^4,5^ with cross section of carbon dioxide varying according to the wavelength of the incident photons as ${10}^{-23} {\text{ cm}}^{2}$ at $195\text{ nm}$, ${10}^{-18}{\text{ cm}}^{2}$ in the range 130–150 nm and ${10}^{-17}{\text{ cm}}^{2}$ in the range 98–120 nm, not allowing penetration of the most energetic region of the ultraviolet (UV) range and much of the X-rays^4,6^. Instead, mid- and near-UV radiations can penetrate the Martian atmosphere, reaching the surface and a few micrometers below the surface, as well as Galactic Cosmic Rays (GCRs) and Solar Energetic Particles (SEPs) which can penetrate even further into the subsurface down to meters^1,7,8^. Even though UV has limited penetration depth in the subsurface^9,10^, it can cause serious damage to organic matter in the aeolian-mobile layer and in the fresh subsurface material exposed to the surface after the abrasions carried out by Perseverance using its abrasion tool to study the interior of the rocks. Many organic molecules absorb UV light and may initiate photochemical reactions undergoing UV-induced degradation in a much shorter time (sols to a few years, depending on the mineral matrix in which the organics are embedded) compared to GCRs and SEPs, whose effects occur in hundreds of millions of years^11–13^. A secondary effect caused by UV that can also damage organics is the UV-induced formation of strong oxidants that may diffuse in the subsurface down to meters and cause oxidative degradation^9,14^.

Perseverance is collecting billion years-old Martian rock cores from a depth of up to about 8 cm below the surface, where organics have been mainly affected by the action of GCRs and SEPs^1^. However, the documentation of the collected samples is performed by characterizing abraded patches of the same rock, which are exposed to ambient Martian UV after the subsurface material is revealed by the Perseverance’s abrasion tool. For rover operational reasons, abraded patches are exposed to ambient UV for at least $1\text{ sol}$ before measuring with proximity science instruments, which can be enough to cause photochemical reactions of possible organics if embedded into photocatalytic mineral matrices^1,15,16^. A severe molecular photodegradation due to exposure to UV before measuring with the Scanning Habitable Environments with Raman and Luminescence for Organics and Chemicals (SHERLOC)^17^. Perseverance instrument might lead to a significant underestimation of the astrobiological relevance of the Jezero samples. SHERLOC, which is a deep UV Raman and fluorescence spectrometer, detected so far the most intense fluorescence features in association with sulfates in Jezero crater^18,19^. There is still a debate on the possible origin of these fluorescence signals, which might be consistent with aromatic organic compounds in association with sulfates or might be due to the presence of rare earth elements in sulfates. The organic hypothesis would be possible only in the presence of organic molecules which remain photostable for at least $1$ sol and/or photoprotection provided by the sulfates. Broz^20^ investigated the organic preservation by sulfates on Earth during terrestrial geological eras and stated that the preservation of organic compounds by sulfur might be a common phenomenon throughout the fossil record of the studied soils. On Earth, sulfate minerals have shown to efficiently trap and preserve organic molecules within their structure^21,22^. Similarly, sulfate minerals might play a role in preserving organic compounds from the oxidizing conditions on Martian surface when trapped within intracrystalline inclusions^23,24^ (as solid inclusions and within fluid inclusions) during some post-depositional alteration, including sulfate mineral growth from saline groundwaters in veins and/or by neomorphic alteration such as recrystallization, which is a well confirmed process in Mars’ past^25^. The extremely slow rates of SO_4_^2−^ reduction would favor the preservation of trapped organics from the harsh Mars surface environmental conditions over extended geological periods^24,26^. Moreover, dos Santos et al.^27^ have shown that sulfates protect amino acids against UV photodamage likely due to their opacity to UV radiation.All these studies suggest that sulfate-rich Martian sediments and rocks can be potential targets for the detection of organic compounds^22^.

In this work, we tested the photostability of aromatic organic molecules in sulfates when exposed to Martian-like UV radiation, in order to verify their probability of surviving at least 1 sol of environmental UV exposure, once revealed by the Perseverance abrasion tool, before SHERLOC analysis. In particular, we investigated hydrated magnesium sulfate because it is widespread in the Jezero samples^28^. A strong Mg-S correlation was observed in the fluorescence data obtained by the Viking landers^29^. Sulfates were subsequently identified as being present in the soil at the Pathfinder landing site at a concentration of ~ $10 \%$ MgSO_4_^30^. Data from the Opportunity rover indicate that sulfates of probable evaporite origin are present in Eagle crater at Meridiani Planum, where the sulfates constitute $\sim 40 \%$ by weight of the bedrock and could have formed by direct precipitation from water or during early burial^31^. In particular, Opportunity’s Miniature Thermal Emission Spectrometer (Mini-TES) data suggest that the major sulfate phases are magnesium and calcium sulfates^32^. Magnesium sulfates were identified also in the Columbia Hills at Gusev Crater, with the Peace class rocks being particularly notable^33^. Additionally, the crystalline magnesium-sulfate mineral starkeyite (MgSO_4_‧$4$H_2_O) was definitively identified using the CheMin X-ray diffraction instrument at Gale crater by Mars Science Laboratory (MSL) Curiosity rover^34^. More specifically, at the Canaima drill site the Mg–sulfate abundance is significant ($\sim 22\%$), composed by starkeyite along with amorphous MgSO_4_‧$n$H_2_O.

Regarding the organic compounds, we investigated aromatic carboxylic acids which are expected to be on Mars since they might be the metastable products of the generic oxidation of meteoritic organic compounds. Experiments show that one of these, benzenehexacarboxylic acid (mellitic acid), is generated by oxidation of organic matter known to arrive to Mars. It is rather stable to further oxidation, and it would not have been easily detected by the Viking experiments^35,36^. Approximately, $2$ kg of meteorite-derived mellitic acid may have been generated per m^2^ of Martian surface over $3$ billion years^37^. Other organic compounds found in meteorites as naphthalene, phenanthrene and anthracene are converted into phthalic acid after photochemical oxidation^38,39^. The oxidation of organics on Mars makes mellitic and phthalic acids appropriate organic compound targets for Martian rover exploration missions. The Curiosity rover of the NASA Mars Science Laboratory mission has already revealed several different organic compounds, including carboxylic acids like phthalic acids, in the MTBSTFA analysis of several samples in Glen Torridon at Gale Crater^40^. In addition, phthalic acid has been indicated as a potential precursor of the previously detected chlorobenzene by the SAM instrument to the Sheepbed mudstone at Gale Crater^26,41^. Furthermore, through the in-situ detection of organic matter preserved in lacustrine mudstones at the base of Murray formation (Gale crater), Eigenbrode et al.^42^ reports that although macromolecules, mineral interactions and permeability factors probably contributed to the preservation of organic matter in the Murray mudstone, sulfurization of organic molecules was probably the main preservation mechanism responsible for the distinct record in the Mojave and Confidence Hills sites, given the presence of 3 to 10 times more thiophene sulfur and total organic sulfur in these samples than in the other mudstones.

We prepared Martian analog samples to simulate a possible natural interaction that might have occurred in an aqueous environment on early Mars between magnesium sulfate and two carboxylic acids, i.e. phthalic acid and mellitic acid, followed by a desiccation event. We characterized the Martian analog samples by InfraRed (IR) from 8000 to 400 cm^−1^ (1.25–25 μm) and Raman spectroscopy from $65$ to $2000{\text{ cm}}^{-1}\, (153-5 \, \mu {\text{m}},$ $532\text{ nm}$ laser source) in order to get insights into the possible molecule-mineral interactions, and compare with data acquired by another instrument on-board Perseverance, namely SuperCam^43^, which includes Raman ($532\text{ nm}$ laser source), Visible and InfraRed (VISIR) reflectance spectroscopy ($400{-}900\text{ nm}$, $1.3{-}2.6 \, \mu {\text{m}}$).

We finally irradiated these analog samples with UV radiation in order to assess the stability of these carboxylic acids when adsorbed on magnesium sulfate once exposed to the ambient Martian UV, and followed the degradation kinetics in situ through infrared spectroscopy.

## Results and discussion

### Phthalic acid IR and Raman characterization: band assignment and spectral changes due to molecular adsorption

**Figure 1:**
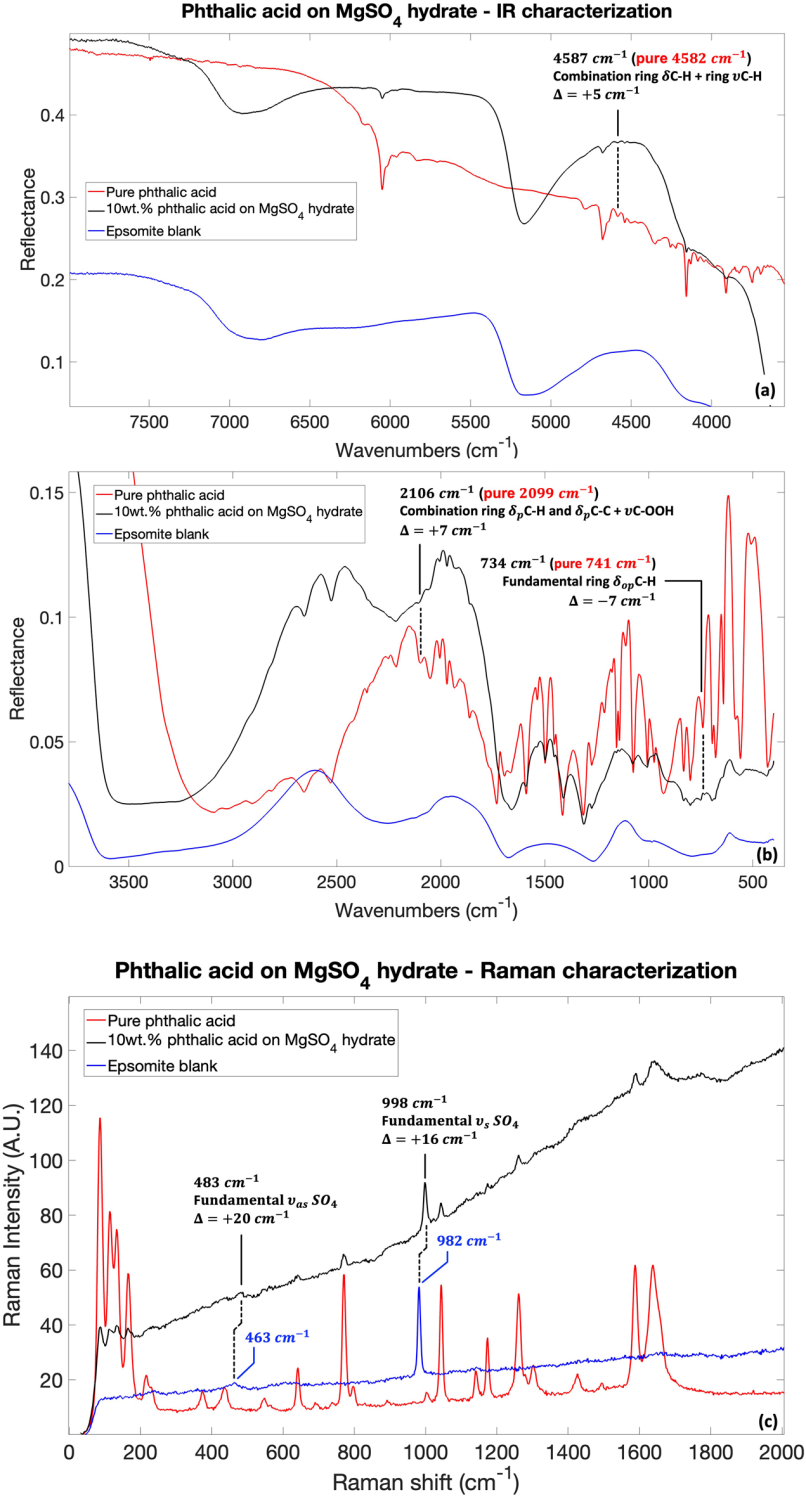
IR and Raman spectra comparison for pure phthalic acid, $10$ wt$\%$ phthalic acid adsorbed on hydrated magnesium sulfate and epsomite blank in (**a**) IR $8000-3500{\text{ cm}}^{-1}$ spectral range; (**b**) IR $3500-500{\text{ cm}}^{-1}$ spectral range; (**c**) Raman $65-2000 \, {\text{cm}}^{-1}$ spectral range. In panels (**a,b**) only adsorbed phthalic acid bands (black spectrum) with shifts greater than the resolution of the instrument ($>4 \, {\text{cm}}^{-1}$) with respect to the pure phthalic acid (red spectrum) are shown. In panel (**c**) the shifts of the hydrated magnesium sulfate (black spectrum) with respect to the epsomite blank $463 \, {\text{cm}}^{-1}$ and $982 \, {\text{cm}}^{-1}$ bands (blue spectrum) are highlighted. Legend: ${\nu }_{s}$ symmetric stretching vibrations; ${\nu }_{as}$ asymmetric stretching vibrations; ${\delta }_{p}$ in-plane bending vibrations; ${\delta }_{op}$ out-of-plane bending vibrations.

Band assignments of the pure carboxylic acid spectra were based on experimental and theoretical data from the literature and on the theoretical anharmonic calculations carried out in this work, especially for the assignment of the combination and overtone bands. Overall, there was a good agreement between the observed band positions and the literature and theoretically calculated ones. As expected, some discrepancies were present because of the differences in the physical state of the samples and/or in the sample preparation methods and spectra acquisition techniques. As already seen in previous works^16,44^, when molecules are adsorbed on the mineral, there is a reduction of both number and intensity of the molecular bands with respect to the pure organic compound. To show the difference in spectral characteristics before and after adsorption, the spectra of pure phthalic acid, $10$ wt% phthalic acid adsorbed on hydrated magnesium sulfate and epsomite blank is shown in Fig. 1. As it can be seen, several vibrational modes of pure phthalic acid are either absent when the phthalic acid is adsorbed on the mineral surface, or the bands are significantly reduced in intensity. Interestingly, regarding IR characterization, the wavenumber range of the spectrum showing more post-adsorption molecular bands are between $2700\text{ and }2500{\text{ cm}}^{-1}\, (3.7{-}4\,\upmu\text{m})$, $2000{-}1900{\text{ cm}}^{-1} \,(5{-}5.3\,\upmu\text{m})$, $1600{-}1300{\text{ cm}}^{-1}$ $(6.25{-}7.7\,\upmu\text{m})$, and $1100{-}600{\text{ cm}}^{-1}, (9.1{-}16.6\,\upmu\text{m})$ (Fig. 1a,b). Outside these spectral ranges, important absorptions of epsomite are present regarding the vibrational modes of SO_4_ and water present in the mineral structure. See Supplementary Table S1 for all the bands detectable both in pure phthalic acid spectrum and phthalic acid adsorbed on hydrated magnesium sulfate with the relative band assignment and intensities.

As it can be appreciated in Supplementary Table S1, almost no shifts of molecular vibrational modes are present when the molecules are adsorbed on magnesium sulfate with respect to the pure molecule. The only three small shifts that can be appreciated are for the pure phthalic acid bands at $4582 \, {\text{cm}}^{-1}$ ($2.2\,\upmu\text{m})$, $2099 \, {\text{cm}}^{-1}$ $(4.8\,\upmu\text{m}$) and $741 \, {\text{cm}}^{-1}\, (13.5\,\upmu\text{m}$), related mainly to ring C–H bending vibrations^45–48^ (Fig. 1a,b). However, the rest of the bands of phthalic acid assigned to the ring C–H vibrations do not present any shifts. It must be highlighted also that the shift of $7 \, {\text{cm}}^{-1}$ or $5 \, {\text{cm}}^{-1}$ are very small taking into account the resolution of the measurements of $4 \, {\text{cm}}^{-1}$. Regarding the kind of molecule-mineral interaction, the incorporation of carboxylic acids within minerals is likely to occur via the creation of ionic bonds between the carboxylic acid group and the mineral surfaces^49^. However, in the adsorbed phthalic acid, none of the bands related mainly to the carboxyl group present any shift with respect to their position in the pure molecule. This could be an indication that the carboxylic acid was not adsorbed on the magnesium sulfate, while it might have been just co-precipitated with the magnesium sulfate during the desiccation process as a dispersed organic material between the magnesium sulfate grains or as intracrystalline inclusions, which would not result in any change of molecular spectroscopic features. Indeed, other studies seem to indicate that when organics are incorporated into sulfate minerals as they grow, their physical, chemical, and spectroscopic properties remain the same. For example, the Raman bands of the organic molecule β-carotene when found as a fluid inclusion inside natural halite^50^ exhibit no shifts with respect to the pure molecule^51–53^. Changes due to a possible interaction with the mineral could also modify the S–O bond length of the sulfate and, as a result, modify the symmetry of the anion, which in the IR spectrum would imply a shift of the bands related to the mineral. However, the sulfate band in the epsomite blank at $1261 \, {\text{cm}}^{-1}$ ($7.9\,\upmu\text{m}$) related to the asymmetric stretching of S$=$O^54–58^ is mostly covered by the bands of phthalic acid when phthalic acid is adsorbed on epsomite. The bands at $1014 \, {\text{cm}}^{-1}$ and $984 \, {\text{cm}}^{-1}$ ($9.9\,\upmu\text{m}$ and $10.2\,\upmu\text{m}$) related to the symmetric stretching of S=O^54–58^ do not change when phthalic acid is adsorbed on epsomite. It is worth noting that the S=O asymmetric stretching band in the epsomite blank and also after the adsorption of the carboxylic acid appears at higher wavenumbers ($1261 \, {\text{cm}}^{-1}$) than expected for pure epsomite around $1100 \, {\text{cm}}^{-1}$ and $1200 \, {\text{cm}}^{-1}$ ($9.1\,\upmu\text{m}$ and $8.3\,\upmu\text{m}$)^54,57,59,60^. According to the work by Lane^61^, studying the IR changes from epsomite to kieserite (MgSO_4_-H_2_O) and to the anhydrous form (MgSO_4_), the sulfate asymmetric stretching shifts to higher wavenumbers with decreasing water content. The same tendency was observed in Raman from gypsum (CaSO_4_-$2$ H_2_O) to bassanite (CaSO_4_-$0.5$H_2_O) to anhydrite (CaSO_4_) by Chio et al.^62^. It seems that the water treatment and subsequent desiccation caused partial dehydration of the original epsomite. The band expected between $615 \, {\text{cm}}^{-1}$ and $620 \, {\text{cm}}^{-1}$ ($16.3\,\upmu\text{m}$ and $16.1\,\upmu\text{m}$) due to the bending mode of the sulfate^54–56,63^ is covered even in the epsomite blank by the broad water libration related band at a bit higher wavenumber around $750 \, {\text{cm}}^{-1}$ ($13.3\,\upmu\text{m})$. Therefore, it is not possible to evaluate any interactions between the organic and the mineral by looking at the IR bands of the sulfate. However, the analysis of the pre- and post-adsorption spectroscopic features of phthalic acid on epsomite reveals a dehydration of the mineral sample with respect to the epsomite blank, and likely multiple hydration state of the magnesium sulfate (MgSO_4_-$n$H_2_O with variable $n$) co-exist in the sample. The dehydration of the initial mineral phase is suggested by the changes in the mineral spectrum of the absorption bands associated with both structural and free water. In particular, the band at $2256 \, {\text{cm}}^{-1}$ ($4.4\,\upmu\text{m}$) in the epsomite blank due to the hydrogen bonded O–H stretching^55,56^ decreases in intensity after the adsorption of the phthalic acid and becomes narrower, as shown in panel (a) of Supplementary Fig. S1, suggesting a dehydration. Hydrogen bonding has a significant influence on this band shape and intensity mainly causing a broadening of the band^56^. Thus, this narrowing might indicate less hydrogen bonds with hydration water molecules maybe as a consequence of an exchange between the organics and the hydration water^56^. The band at $3583 \, {\text{cm}}^{-1}$ ($2.8\,\upmu\text{m}$), assigned to O–H stretching^63^, shows substantial changes. Usually, this band has a lower wavenumber shoulder when seven molecules are present in the structure (MgSO_4_-$7$H_2_O, epsomite)^64^. However, in the spectrum of phthalic acid adsorbed on epsomite this shoulder is reduced (dashed red arrows in panel (b) of Supplementary Fig. S1), and an $88 \, {\text{cm}}^{-1}$ shift to lower wavenumbers (red-shift) is observed, which is consistent with dehydration, as shown by Bonello et al.^64^. The O–H stretching and O–H bending combination band^65^ at $5163 \, {\text{cm}}^{-1}$ ($1.9\,\upmu\text{m}$) also undergoes some changes, specifically a narrowing and a reduction of the shoulder (dashed red arrows) as already observed for the band at $3583 \, {\text{cm}}^{-1}$ (Panel (c) in Supplementary Fig. S1), along with a blueshift of $6 \, {\text{cm}}^{-1}$ in the spectrum of phthalic acid adsorbed on epsomite, consistent with epsomite dehydration^64^. Finally, another proof of dehydration comes from the band assigned to the $1$st overtone of the O–H stretching^65^ at $6792 \, {\text{cm}}^{-1}$ ($1.5\,\upmu\text{m}$) which undergoes a shift of $130 \, {\text{cm}}^{-1}$ to higher wavenumbers (blue-shift) and a considerable narrowing when phthalic acid is adsorbed on epsomite, as shown in panel (d) of Supplementary Fig. S1. Both water bands and the position of the bending vibrational mode of the sulfate indicate a dehydration of the original epsomite. This dehydration may be partially due to the replacement of free water molecules in the epsomite structure with phthalic acid molecules as a consequence of molecular inclusion, but likely there is also an effect of dehydration due to the desiccation process since the bending vibrational mode of the sulfate in the blank is also at higher wavenumbers than the one expected for epsomite. This band after the molecular adsorption, however, is difficult to compare with the blank due to the presence of many bands belonging to the phthalic acid within it. In summary, the changes in the IR spectrum of phthalic acid adsorbed on magnesium sulfate suggest a mineral dehydration from MgSO_4_-$7$H_2_O (epsomite) to MgSO_4_-$n$H_2_O with $2\le n\le 6$, according to Bonello et al.^64^.

Raman spectroscopy results show that the bands associated with the phthalic acid molecule are attenuated but still visible in the adsorbed case, without undergoing appreciable shifts, in agreement with infrared spectroscopy results. The epsomite bands at $982 \, {\text{cm}}^{-1}$ and $463 \, {\text{cm}}^{-1}$ ($10.2\,\upmu\text{m}$ and $21.6\,\upmu\text{m}$), assigned to the SO_4_ symmetric and asymmetric stretching vibrations^66^, undergo a blue-shift of $16 \, {\text{cm}}^{-1}$ and $20 \, {\text{cm}}^{-1}$, respectively, in the presence of the phthalic acid molecule (Fig. 1c), moving towards $998 \, {\text{cm}}^{-1}$ and $483 \, {\text{cm}}^{-1}$ ($10\,\upmu\text{m}$ and $20.7\,\upmu\text{m}$). Comparing this result with the dehydration studies of magnesium sulfate by Wang et al.^66^, there is a shift from a structure with seven water molecules (MgSO_4_-$7$H_2_O, epsomite) to one with four (MgSO_4_-$4$H_2_O, starkeyite) or five water molecules (MgSO_4_-5H_2_O, pentahydrite), in agreement with IR measurements.

### Mellitic acid IR and Raman characterization: band assignment and spectral changes due to molecular adsorption

**Figure 2:**
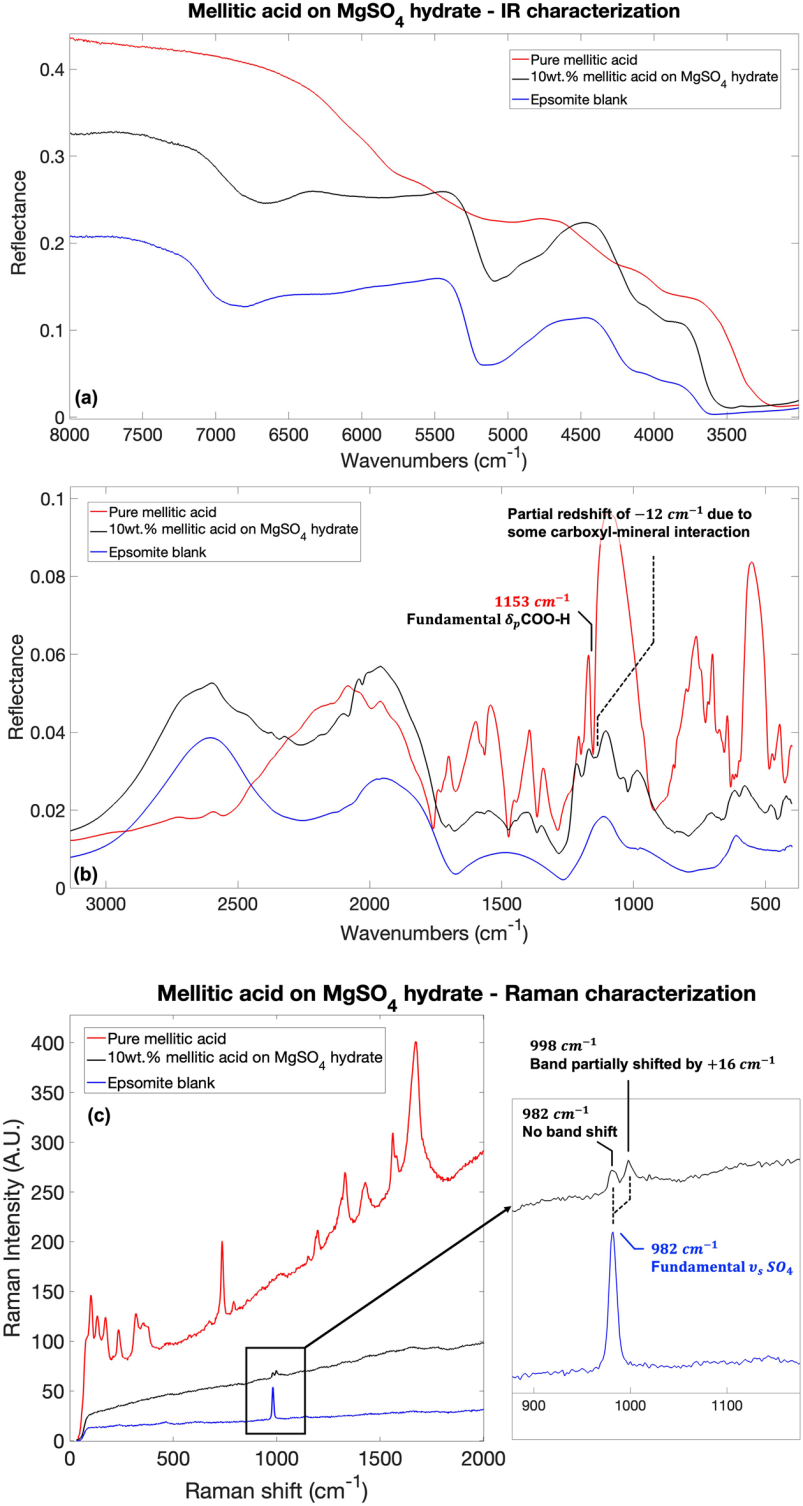
IR and Raman spectra comparison for pure mellitic acid, $10$ wt$\%$ mellitic acid adsorbed on hydrated magnesium sulfate and epsomite blank in (**a**) IR $8000{-}3500 \, {\text{cm}}^{-1}$ spectral range; (**b**) IR $3500{-}500 \, {\text{cm}}^{-1}$ spectral range; (**c**) Raman $65{-}2000 \, {\text{cm}}^{-1}$ spectral range. In panels (**b**) the partial redshift of the $1153 \, {\text{cm}}^{-1}$ band due to interaction between mellitic acid and hydrated magnesium sulfate via carboxyl groups is shown. In panel (**c**) a partial blueshift of the $982 \, {\text{cm}}^{-1}$ band due to the mineral dehydration is shown. Legend: ${\nu }_{s}$ symmetric stretching vibrations; ${\delta }_{p}$ in-plane bending vibrations$.$

To show the difference in spectral characteristics before and after adsorption, the spectra of pure mellitic acid, $10$ wt$\%$ mellitic acid adsorbed on hydrated magnesium sulfate and epsomite blank is shown in Fig. 2.

As in the phthalic acid case, several vibrational modes of pure mellitic acid are either absent when the mellitic acid is adsorbed on the mineral, or the bands are significantly reduced in intensity. Interestingly, regarding IR characterization, the wavenumber range of the spectrum showing more post-adsorption molecular bands are between $1600\text{ and }1300 \, {\text{cm}}^{-1}\, (6.3{-}7.7\,\upmu\text{m})$ and $1200{-}1000 \, {\text{cm}}^{-1} \, (8.3{-}10\,\upmu\text{m})$. Outside these spectral ranges, important absorptions of epsomite are present regarding the vibrational modes SO_4_ and water present in the mineral structure. See Supplementary Table S2 for all the bands detectable both in pure mellitic acid spectrum and mellitic acid adsorbed on magnesium sulfate with the relative band assignment and intensities. Almost no shifts of molecular vibrational modes are present when the molecules are adsorbed on magnesium sulfate with respect to the pure molecule. The only remarkable change concerns the splitting of the $1153 \, {\text{cm}}^{-1}$ ($8.7\,\upmu\text{m}$) band of mellitic acid when adsorbed on epsomite, which is associated mainly with the fundamental COO–H in-plane bending vibration of the COOH group as observed through computational spectroscopy simulations. Such a splitting might indicate that part of the mellitic acid molecules interact with the sulfate through the carboxyl functional group giving rise to the new band redshifted of $12 \, {\text{cm}}^{-1}$ that appears when mellitic acid is adsorbed on epsomite (Fig. 2b).

As in the case of phthalic acid, there is evidence of spectral changes in the spectrum of mellitic acid adsorbed on epsomite attributable to dehydration of epsomite. The presence of multiple sulfate hydration phases is suggested by the $984 \, {\text{cm}}^{-1}$ ($10.2\,\upmu\text{m}$) band blue-shift to $997 \, {\text{cm}}^{-1}$ ($10\,\upmu\text{m}$) and the clear appearance of two bands at $1051 \, {\text{cm}}^{-1}$ and $1022 \, {\text{cm}}^{-1}$ ($9.5\,\upmu\text{m}$ and $9.8\,\upmu\text{m}$), previously convoluted under one broader band centered at $1030 \, {\text{cm}}^{-1}$ ($9.7\,\upmu\text{m}$), associated with symmetric sulfate vibrations^54–58^, as shown in panel (a) of Supplementary Fig. S2, that might be a consequence of different bonding with structural water molecules. Moreover, the band associated with water O–H stretching^63^ at $3583 \, {\text{cm}}^{-1}$ ($2.8\,\upmu\text{m}$) undergoes a spectral shift of $114 \, {\text{cm}}^{-1}$, which further suggests dehydration in agreement with Bonello et al.^64^ (Panel (c) of Supplementary Fig. S2)*.* Then, the band at $5163 \, {\text{cm}}^{-1}$ ($1.9\,\upmu\text{m}$) assigned to the combination of water O–H stretching and O–H bending^65^ presents a redshift of $71 \, {\text{cm}}^{-1}$ after the adsorption of mellitic acid, consistent with a stronger dehydration of the original epsomite to a hydrated magnesium sulfate MgSO_4_-$n$H_2_O with $1\le n\le 5$, according to Bonello et al.^64^ (Panel (d) of Supplementary Fig. S2). Thus, multiple hydration states of magnesium sulfate should co-exist in this sample. Finally, further evidence of a high dehydration can be appreciated from the band at $6792 \, {\text{cm}}^{-1}$ ($1.5\,\upmu\text{m}$) assigned to the $1$st overtone of the water O–H stretching^65^, which presents a redshift of $133 \, {\text{cm}}^{-1}$ in the post-adsorption as shown in panel (e) of Supplementary Fig. S2, consistent with a stronger dehydration associated with the presence of kieserite MgSO_4_-H_2_O^64^. In summary, the changes in the IR spectrum of mellitic acid adsorbed on magnesium sulfate suggest a molecular interaction through the carboxyl groups, which was not observed for the phthalic acid, and stronger dehydration of the magnesium sulfate than when the phthalic acid is adsorbed on epsomite. See Supplementary Fig. S2 for the spectral changes in the spectrum of mellitic acid adsorbed on epsomite attributable to dehydration of epsomite.

Raman spectroscopy results show that the molecular bands associated with mellitic acid are strongly attenuated or absent once the molecule is adsorbed onto the mineral. A part of the bonds constituting the epsomite band at $982 \, {\text{cm}}^{-1}$ ($10.2\,\upmu\text{m}$), assigned to the SO_4_ stretching vibration^66^, undergoes a blue-shift of $16 \, {\text{cm}}^{-1}$ in the presence of the mellitic acid molecule (Fig. 2c), moving to $998 \, {\text{cm}}^{-1}$ ($10\,\upmu\text{m}$). Comparing this result with the dehydration studies of magnesium sulfate by Wang et al.^66^, there is a shift from a structure with seven water molecules (MgSO_4_-$7$H_2_O, epsomite) to one with four (MgSO_4_-$4$H_2_O, starkeyite) or five water molecules (MgSO_4_-$5$H_2_O, pentahydrite), in agreement with IR measurements.

### UV irradiation of pure phthalic acid and $10$ wt% phthalic acid on hydrated magnesium sulfate

Table 1 reports the bands analyzed for the study of the photodegradation kinetics of pure phthalic acid with the corresponding assignments based on DFT calculations carried out in this work and literature^45–48^, degradation rate ($\beta$), half-lives (${t}_{1/2}$**)** and destruction cross section ($\upsigma$) results from the fit model described in the Methods section. The fourth column is the half-life assuming dust free atmosphere at the noontime equator according to Patel et al.^5^. Instead, the fifth column is the half-life according to annual mean UV flux at the surface at Jezero crater. See Supplementary Table S3 for the remaining fitting parameters.

**Table 1:** Degradation rate ($\beta$), half-lives (${t}_{1/2}$) and destruction cross section ($\upsigma$) for pure phthalic acid, along with vibrational mode assignment indicating in bold the main vibration.

Band (cm^−1^)	Phthalic acid vibrational mode	$\beta\, [{\text{s}}^{-1}]$	${t}_{1/2}\, \left[{\text{sol}}\right]$ Patel et al. (2002) flux	${t}_{1/2} \,[{\text{sol}}]$ Jezero crater flux	$\sigma \,[{\text{cm}}^{2}]$
4046	Combination **C=O stretching** + COO-H in-plane bending + ring C–H in-plane bending	$\left(1.1\pm 0.5\right)\times {10}^{-2}$	$0.3\pm 0.1$	$0.4\pm 0.2$	$\left(4\pm 2\right)\times {10}^{-20}$
2423	Combination ring C–H in-plane bending + COOH bending	$\left(6\pm 3\right)\times {10}^{-4}$	$5\pm 3$	$7\pm 4$	$\left(2\pm 1\right)\times {10}^{-21}$
1969	1^st^ overtone **ring C**–**H out-of-plane bending** and ring C–C out-of-plane bending	$\left(8\pm 1\right)\times {10}^{-4}$	$4\pm 1$	$5\pm 1$	$\left(2.8\pm 0.5\right)\times {10}^{-21}$
1693	Fundamental carboxyl C=O stretching	$\left(8\pm 2\right)\times {10}^{-3}$	$0.4\pm 0.1$	$0.5\pm 0.2$	$\left(3\pm 1\right)\times {10}^{-20}$
1275	Fundamental carboxyl C–O stretching	$\left(4.4\pm 0.8\right)\times {10}^{-3}$	$0.7\pm 0.1$	$0.9\pm 0.2$	$\left(1.6\pm 0.3\right)\times {10}^{-20}$
1213	Combination COOH bending + ring C–H and C–C out-of-plane bending	$\left(1.8\pm 0.4\right)\times {10}^{-3}$	$1.8\pm 0.4$	$2.3\pm 0.5$	$\left(7\pm 1\right)\times {10}^{-21}$
1155	Fundamental ring C–H bending	$\left(1.3\pm 0.4\right)\times {10}^{-4}$	$24\pm 8$	$30\pm 10$	$\left(5\pm 2\right)\times {10}^{-22}$
1142	Fundamental ring C–H in-plane bending	$\left(2.4\pm 0.5\right)\times {10}^{-4}$	$13\pm 3$	$17\pm 3$	$\left(9\pm 2\right)\times {10}^{-22}$
1111	Fundamental ring C–H in-plane bending	$\left(2\pm 1\right)\times {10}^{-4}$	$15\pm 8$	$20\pm 10$	$\left(7\pm 4\right)\times {10}^{-22}$
1074	Fundamental ring C–C in-plane bending	$\left(3.3\pm 0.7\right)\times {10}^{-4}$	$9\pm 2$	$12\pm 3$	$\left(1.2\pm 0.2\right)\times {10}^{-21}$
1007	Fundamental ring C–H out-of-plane bending	$\left(3.3\pm 0.8\right)\times {10}^{-4}$	$9\pm 2$	$13\pm 3$	$\left(1.2\pm 0.3\right)\times {10}^{-21}$
974	Fundamental ring C–H out-of-plane bending	$\left(3\pm 1\right)\times {10}^{-4}$	$10\pm 4$	$14\pm 5$	$\left(1.1\pm 0.4\right)\times {10}^{-21}$
800	Fundamental ring C–H and C–C out-of-plane bending	$\left(5\pm 2\right)\times {10}^{-4}$	$6\pm 3$	$8\pm 3$	$\left(2\pm 1\right)\times {10}^{-21}$
741	Fundamental ring C–H out-of-plane bending	$\left(2.0\pm 0.9\right)\times {10}^{-4}$	$15\pm 7$	$20\pm 7$	$\left(8\pm 3\right)\times {10}^{-22}$

The fourth column is the half-life values assuming dust free atmosphere at the noontime equator according to Patel et al.^5^. Instead, the fifth column is the half-life values according to the annual mean UV flux at Jezero crater. See Supplementary Table S3 for the remaining fitting parameters.

**Figure 3:**
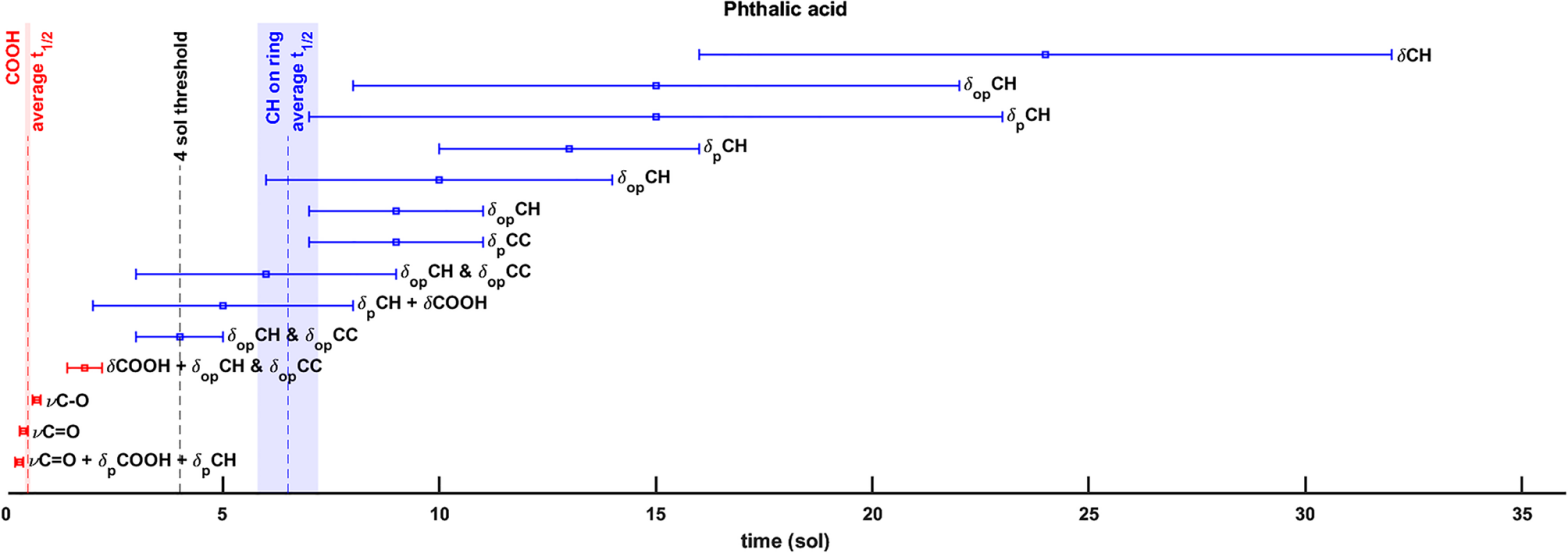
Pure phthalic acid half-lives degradation values $({\text{sol}})$ according to Patel et al.^5^ UV flux. A $4\text{ sol}$ threshold value can be identified between the bands mainly assigned to the COOH carboxyl group (red) and the bands mainly assigned to ring C–H bands (blue) degradations. Legend: $\nu$ stretching vibrations; ${\delta }_{p}$ in-plane bending vibrations; ${\delta }_{op}$ out-of-plane bending vibrations.

As shown in Table 1, according to Patel et al.^5^ UV flux, the combination mainly assigned to C=O stretching $4046 \, {\text{cm}}^{-1}$ ($2.5\,\upmu\text{m}$) and the fundamental carboxyl C$=$O stretching $1693 \, {\text{cm}}^{-1}$ ($5.9\,\upmu\text{m}$) bands, present the fastest degradations: ${t}_{1/2}=(0.3\pm 0.1)\text{ sol}$ and ${t}_{1/2}=$ ($0.4\pm 0.1)\text{ sol}$, respectively, suggesting that the carboxyl group degrades first during irradiation. The degradation of the carboxyl groups is also observed in the decrease of other two bands: the fundamental carboxyl C–O stretching $1275 \, {\text{cm}}^{-1}$ ($7.8\,\upmu\text{m}$) and the combination COOH + ring C–H and C–C out-of-plane bending $1213 \, {\text{cm}}^{-1}$ ($8.2\,\upmu\text{m}$) bands with ${t}_{1/2}=$ ($0.7\pm 0.1)\text{ sol}$ and ${t}_{1/2}=(1.8\pm 0.4)\text{ sol}$, respectively. The latter band, $1213 \, {\text{cm}}^{-1}$, has slightly higher half-life than the $4046 \, {\text{cm}}^{-1}$, $1693 \, {\text{cm}}^{-1}$ and $1275 \, {\text{cm}}^{-1}$ bands, which are associated only or mainly to COOH carboxyl group bonds. This is likely due to the contribution of the ring aromatic ring in such vibrational mode, and then less strongly from the COOH vibrations, as observed through computational simulations. This contribution slows down the degradation of the band and thus increases the overall half-life, as shown in Fig. 3. See panels a,d–f of Supplementary Fig. S3 for the curve fits mentioned above.

Beyond the range of degradation times associated with the carboxyl group, we find higher half-lives for the absorption bands attributable to the aromatic ring zone. In particular, the assignments of such bands seem to be associated more with the in-plane and out-of-plane bending vibrations of the aromatic ring C–H bonds as shown in Table 1. The band at $1969 \, {\text{cm}}^{-1}$ ($5.1\,\upmu\text{m}$), assigned mainly to the ring C–H out-of-plane bending, has a half-life of ${t}_{1/2}=\left(4\pm 1\right)\text{ sol}$. This value of approximately $4\text{ sol}$ turns out to be a threshold degradation time between the half-life of the COOH carboxyl group (${t}_{1/2}\lesssim 4\text{ sol}$) and the aromatic ring (${t}_{1/2}\gtrsim 4\text{ sol}$) of the molecule (Fig. 3). Above the value of $4 sol$, consistent photodegradations were obtained for the bands at $2423 \, {\text{cm}}^{-1}\, ($4.1 $\,\upmu\text{m}$), $1969 \, {\text{cm}}^{-1}\, (5.1\,\upmu\text{m})$, $1155 \, {\text{cm}}^{-1}\, (8.7\,\upmu\text{m})$, $1142 \, {\text{cm}}^{-1}\, (8.8\,\upmu\text{m})$, $1111 \, {\text{cm}}^{-1}\, (9.0\,\upmu\text{m})$, $1074 \, {\text{cm}}^{-1}\, (9.3\,\upmu\text{m})$, $1007 \, {\text{cm}}^{-1}\, (9.9\,\upmu\text{m})$, $974 \, {\text{cm}}^{-1}\, (10.3\,\upmu\text{m})$, $800 \, {\text{cm}}^{-1}\, (12.5\,\upmu\text{m})$ and $741 \, {\text{cm}}^{-1}\, (13.5\,\upmu\text{m})$ with half-lives $4\lesssim {t}_{1/2}\lesssim 24\text{ sol}$ and a maximum measurement error between $40$ and $50 \%$ recorded for the $2423$, $1111$, $974$, $800$ and $741 \, {\text{cm}}^{-1}$ bands (for the latter two due to the low SNR) and error of $30 \%$ or less for the others as shown in Fig. 3. See panels b,c,g–n of Supplementary Fig. S3 for all the fit curve plots.

**Figure 4:**
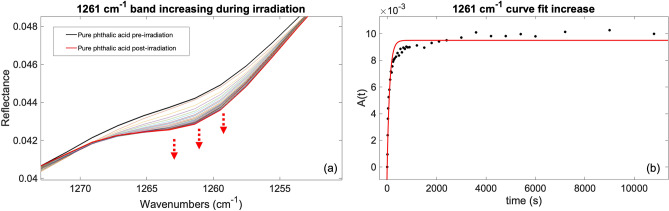
Pure phthalic acid $1261 \, {\text{cm}}^{-1}$ $(7.9\,\upmu\text{m})$ band increase: (**a**) IR spectra changing during irradiation experiment; (**b**) curve fit for the $1261 \, {\text{cm}}^{-1}$ band.

In addition, as shown in Fig. 4, the appearance of a new band at $1261 \, {\text{cm}}^{-1}\, (7.9\,\upmu\text{m})$, was observed during UV irradiation. The formation rate for this band is $\alpha =\left(9.7\pm 0.8\right)\times {10}^{-3}{\text{ s}}^{-1}$ while the formation cross section is ${\upsigma }_{f}=\left(3.5\pm 0.3\right)\times {10}^{-20} \, {\text{cm}}^{2}$, which is consistent with the degradation rates of the carboxylic COOH groups (Table 2). The same new band at $1261 \, {\text{cm}}^{-1}$ was also observed in the mellitic acid irradiation experiment, due to a photoreaction involving the rotation of the carboxyl group followed by the interaction of neighboring hydroxyl groups resulting in the formation of an anhydrous group (see Fig. 7). Since phthalic acid has two adjacent carboxyl groups in its molecular structure, it is plausible that the same photoreaction occurs. The presence of another new band in the phthalic acid spectrum at $1383 \, {\text{cm}}^{-1}$ (again, the same position as the new band observed in the mellitic acid irradiation) corroborates this hypothesis; however, it cannot be integrated due to its low intensity (Supplementary Fig. S4).

**Table 2:** Pure and adsorbed phthalic acid photoproduct results: formation rate ($\alpha$) and formation cross section (${\upsigma}_{f}$).

Band [${\text{cm}}^{-1}$]	Pure phthalic acid results		Adsorbed phthalic acid results	
	$\alpha\, [{\text{s}}^{-1}]$	${\upsigma }_{f} \,[{\text{cm}}^{2}]$	$\alpha \,[{\text{s}}^{-1}]$	${\upsigma }_{f} \,[{\text{cm}}^{2}]$
1383	Not integrable	Not integrable	$\left(1.0\pm 0.3\right)\times {10}^{-3}$	$\left(3.6\pm 0.9\right)\times {10}^{-21}$
1261	$\left(9.7\pm 0.8\right)\times {10}^{-3}$	$\left(3.5\pm 0.3\right)\times {10}^{-20}$	Not available	Not available

Based on these degradation times, we hypothesize that degradation of pure phthalic acid starts from the carboxyl functional groups with a weighted average half-life of $\overline{{t}_{1/2}^{\text{COOH}}}=(0.49\pm 0.06)\text{ sol}$. Once passed the threshold of $4$ sols of irradiation, the degradation proceeds to the C–H of the aromatic ring with a weighted average half-life of $\overline{{t}_{1/2}^{ ring {\text{CH}}}}=(6.5\pm 0.7)\text{ sol}$. The aromatic ring does not degrade, rather the intensity of two bands at $1589 \, {\text{cm}}^{-1}$ and $1535 \, {\text{cm}}^{-1}$, both assigned to the fundamental benzene C–C stretching increases, as shown in Supplementary Fig. S6, which suggests an increase of aromaticity of the molecules as a consequence of UV irradiation. Overall, the half-lives found in our work for pure phthalic acid are in good agreement with the ones reported by Noblet et al.^67^.

In the case of $10$ wt$\%$ phthalic acid adsorbed on hydrated magnesium sulfate, instead, despite the observation of strong molecular bands, no appreciable photodegradation was recorded. Often, an initial signal increase was obtained for the molecular bands followed by a horizontal plateau. The increase at the beginning can be associated with a change in the continuum of the spectrum around the band under investigation during the first moments of the irradiation experiment. The plateau observed afterwards indicates that no changes followed this effect with the UV irradiation time. In addition, the same new band at $1383 \, {\text{cm}}^{-1}$ observed in the pure phthalic acid case appears also when phthalic acid is adsorbed on magnesium sulfate indicating the occurrence of the same photoreaction described above (Supplementary Fig. S5). The formation rate of this band is $\alpha =\left(1.0\pm 0.3\right)\times {10}^{-3}{\text{ s}}^{-1}$ with a formation cross section of ${\upsigma }_{f}=\left(3.6\pm 0.9\right)\times {10}^{-21}{\text{ cm}}^{2}$. The estimated formation rates are reported in Table 2.

### UV irradiation of pure mellitic acid and $10$ wt% mellitic acid on hydrated magnesium sulfate

**Figure 5:**
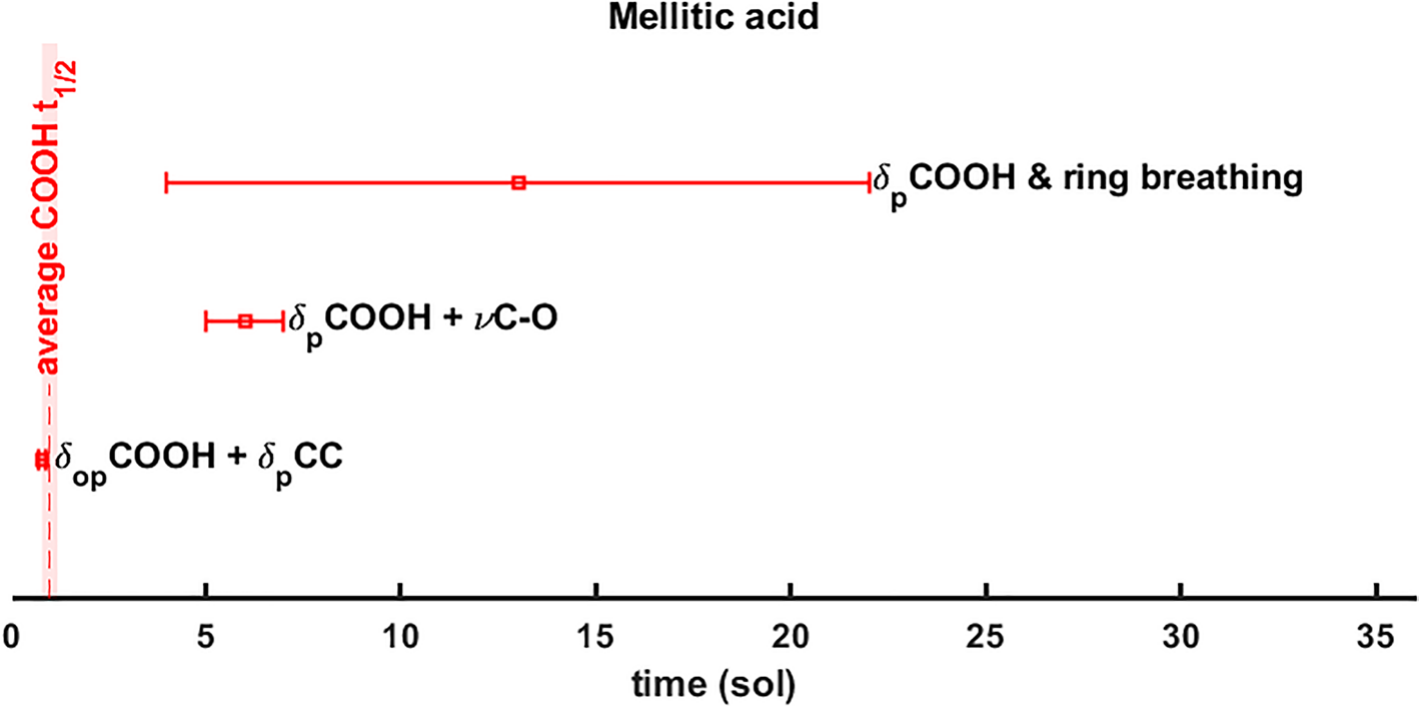
Pure mellitic acid half-life degradation values $({\text{sol}})$ according to Patel et al.^5^ UV flux for the bands mainly assigned to the COOH carboxyl group. Legend: $\nu$ stretching vibrations; ${\delta }_{p}$ in-plane bending vibrations; ${\delta }_{op}$ out-of-plane bending vibrations.

In contrast with pure phthalic acid, most of the bands of pure mellitic acid do not degrade during UV irradiation. Only three vibrational bands exhibit a decrease in intensity: the combination mainly assigned to COO-H out-of-plane bending at ${1564\text{ cm}}^{-1}$ ($6.4\,\upmu\text{m}$), the fundamental COOH in-plane bending and ring breathing at ${1366\text{ cm}}^{-1}$ ($7.3\,\upmu\text{m}$) and the fundamental COO-H in-plane bending and C–O stretching band at ${1153\text{ cm}}^{-1}$ ($8.7\,\upmu\text{m}$) bands. This indicates that only the part of the molecule involving the carboxyl group is affected by UV. The curve fit degradations are shown in Fig. S7. The half-lives obtained are ${t}_{1/2}=(0.8\pm 0.2)\text{ sol}$ for the ${1564\text{ cm}}^{-1}$ band, ${t}_{1/2}=(13\pm 9)\text{ sol}$ for $1366 \, {\text{cm}}^{-1}$ and ${t}_{1/2}=(6\pm 1)\text{ sol}$ for ${1153\text{ cm}}^{-1}$ as shown in Table 3, which allow us to estimate a weighted average half-life of $\overline{{t}_{1/2}^{\text{COOH}}}=(1.0\pm 0.2)\text{ sol}$ for the COOH carboxyl groups (Fig. 5). These results are in good agreement with previous studies by Stalport et al.^68^. See Supplementary Table S4 for the remaining fit parameters.

**Table 3:** Degradation rate ($\beta$), half-lives (${t}_{1/2}$) and destruction cross section ($\upsigma$) for pure mellitic acid, along with vibrational mode assignment indicating in bold the main vibration.

Band [${\text{cm}}^{-1}$]	Mellitic acid vibrational mode	$\beta \,[{\text{s}}^{-1}]$	${t}_{1/2}\, \left[{\text{sol}}\right]$ Patel et al.^5^	${t}_{1/2}\, \left[{\text{sol}}\right]$ Jezero crater flux	$\sigma \,[{\text{cm}}^{2}]$
1564	Combination **COO-H out-of-plane bending** + ring C–C in-plane bending	$\left(4\pm 1\right)\times {10}^{-3}$	$0.8\pm 0.2$	$1.0\pm 0.3$	$\left(1.5\pm 0.4\right)\times {10}^{-20}$
1366	Fundamental COOH in-plane bending and ring breathing	$\left(2.4\pm 1.8\right)\times {10}^{-4}$	$13\pm 9$	$17\pm 12$	$\left(9\pm 6\right)\times {10}^{-22}$
1153	Fundamental **COO-H in-plane bending** and C–O stretching	$\left(5\pm 1\right)\times {10}^{-4}$	$6\pm 1$	$8\pm 2$	$\left(1.9\pm 0.4\right)\times {10}^{-21}$

The fourth column is the half-lives value assuming dust free atmosphere at the noontime equator according to Patel et al.^5^. Instead, the fifth column is the half-life values according to the annual mean UV flux at Jezero crater. See Supplementary Table S4 for the remaining fitting parameters.

**Figure 6:**
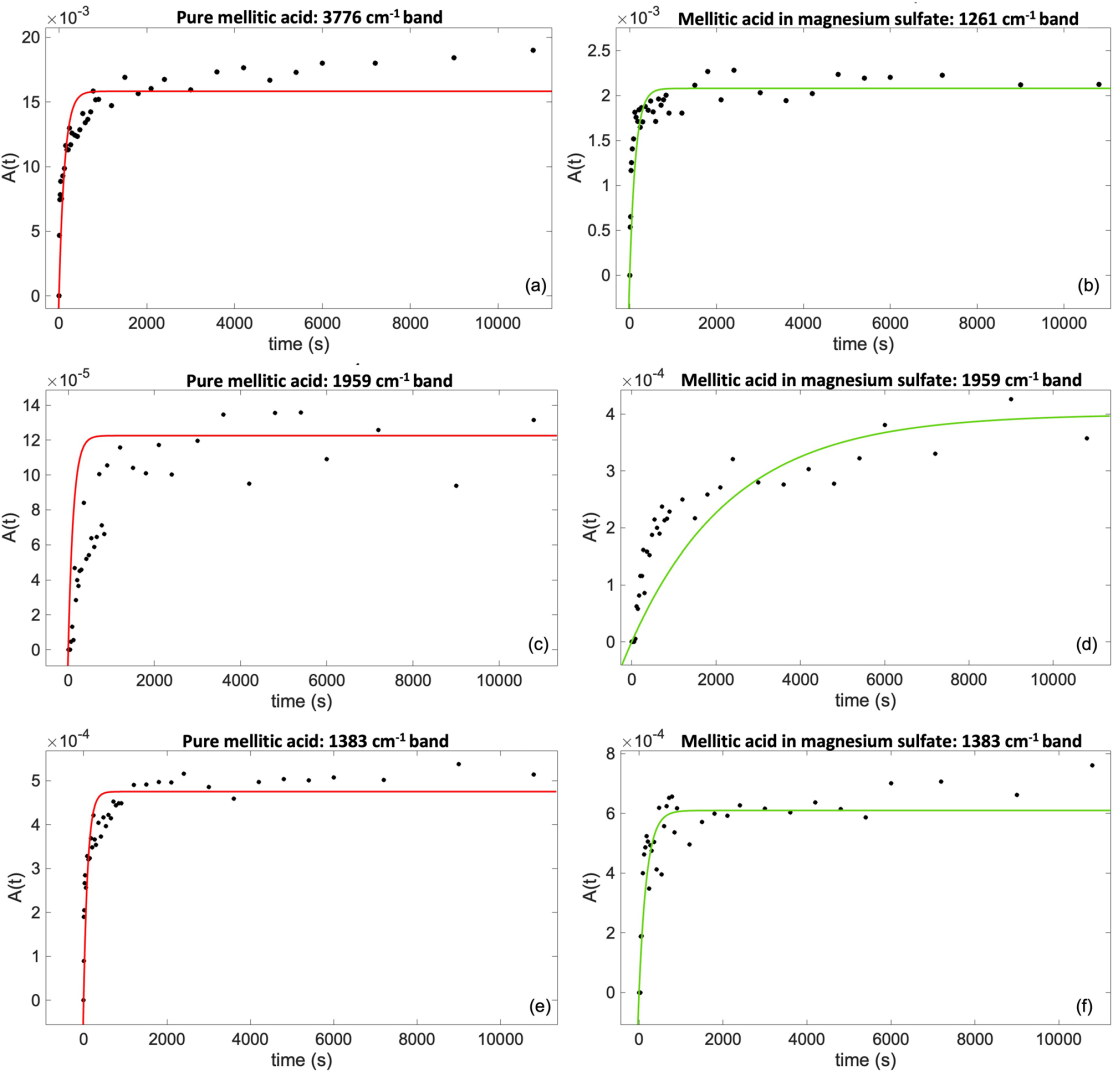
Fit model (red for pure mellitic acid and green for adsorbed mellitic acid) regarding the formation of the photoproduct bands for (**a**) pure mellitic acid $3776 \, {\text{cm}}^{-1}$; (**b**) adsorbed mellitic acid $1261 \, {\text{cm}}^{-1}$; (**c**) pure mellitic acid $1959 \, {\text{cm}}^{-1}$; (**d**) adsorbed mellitic acid $1959 \, {\text{cm}}^{-1}$; (**e**) pure mellitic acid $1383 \, {\text{cm}}^{-1}$; (**f**) adsorbed mellitic acid $1383 \, {\text{cm}}^{-1}$.

Moreover, during UV irradiation of pure mellitic acid, four new bands due to formation of photoproducts appear at $3776 \, {\text{cm}}^{-1}$ ($2.7\,\upmu\text{m}$) (Fig. 6a), $1959 \, {\text{cm}}^{-1}$ ($5.1\,\upmu\text{m}$) (Fig. 6c), $1383 \, {\text{cm}}^{-1}$ ($7.2\,\upmu\text{m}$) (Fig. 6e) and ${1261\text{ cm}}^{-1}$ ($7.9\,\upmu\text{m}$). The estimated formation rates are reported in Table 4.

**Table 4:** Pure and adsorbed mellitic acid photoproduct results: formation rate ($\alpha$) and formation cross section (${\upsigma}_{f}$).

Band [${\text{cm}}^{-1}$]	Pure mellitic acid results		Adsorbed mellitic acid results	
	$\alpha\, [{\text{s}}^{-1}]$	${\upsigma }_{f} \,[{\text{cm}}^{2}]$	$\alpha\, [{\text{s}}^{-1}]$	${\upsigma }_{f} \,[{\text{cm}}^{2}]$
3776	$(8\pm 1)\times {10}^{-3}$	$(2.9\pm 0.4)\times {10}^{-20}$	Not available	Not available
1959	$(8\pm 3)\times {10}^{-3}$	$(3\pm 1)\times {10}^{-20}$	$(1.1\pm 0.1)\times {10}^{-3}$	$(4.0\pm 0.4)\times {10}^{-21}$
1404	Not available	Not available	Not integrable	Not integrable
1383	$\left(1.0\pm 0.2\right)\times {10}^{-2}$	$(3.6\pm 0.7)\times {10}^{-20}$	$(5.5\pm 0.8)\times {10}^{-3}$	$(2.0\pm 0.3)\times {10}^{-20}$
1261	Not integrable	Not integrable	$(7\pm 1)\times {10}^{-3}$	$(2.6\pm 0.4)\times {10}^{-20}$

**Figure 7:**
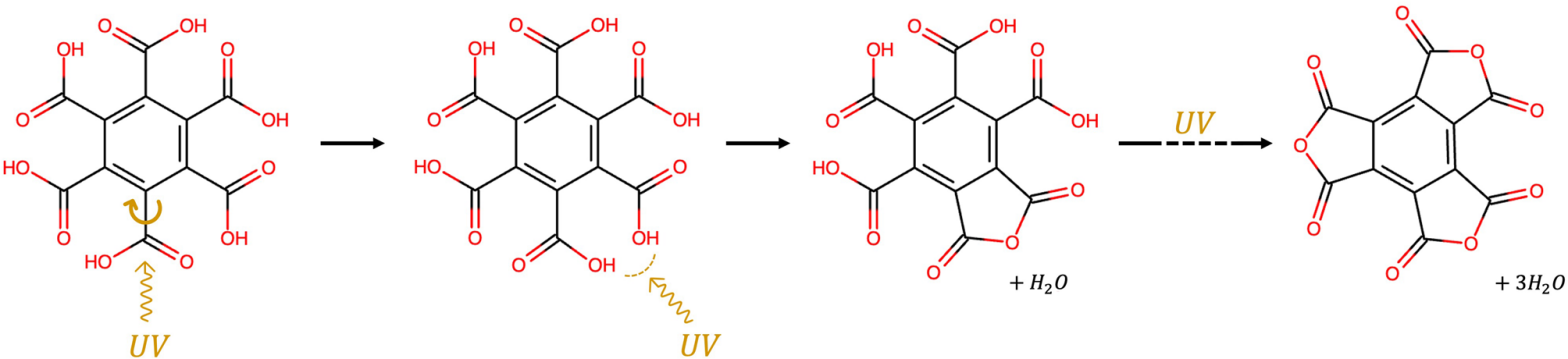
Intramolecular pathway for the formation of benzenehexacarboxylic acid-trianhydride from mellitic acid proposed by Stalport et al.^69^ (adapted from Stalport et al.^69^).

These formation rates have the same order of magnitude of the degradation rate of the combination band mainly assigned to COO-H out-of-plane bending at ${1564\text{ cm}}^{-1}$, while are an order of magnitude higher than the degradation rates of the bands at $1366 \, {\text{cm}}^{-1}$ associated to fundamental COOH bending and ring breathing and $1153 \, {\text{cm}}^{-1}$ associated mainly to fundamental COO-H bending. These results suggest that the COO-H bond of the carboxyl group plays a key role in the mellitic acid photochemistry, which is consistent with previous studies by Stalport et al.^69^ showing that after $100$ hours of UV irradiation mellitic acid undergoes photo-transformation into a UV-resistant compound identified as benzenehexacarboxylic acid-trianhydride (${\text{C}}_{12}{\text{O}}_{9}$), thanks to a reaction involving the COOH group^69^. The photoreaction pathway involves intramolecular chemical reactions, where the energy provided by UV light would cause the rotation of COOH carboxyl groups resulting in the catalysis of the interaction between neighboring O–H groups producing anhydrous groups (Fig. 7). One of the non-bonding electron pairs of an oxygen atom of a first O–H group forms a connection with the proton of a second neighboring O–H group, forming three non-bonding electron pairs on the oxygen atom that has lost its proton. One of these electron pairs would form a connection with a carbon atom that has an electron deficit. This intramolecular reaction would form an oxygen bond between two carbon atoms and a free water molecule as shown in Fig. 7.

Another possible pathway could involve intermolecular chemical reactions, where two neighboring O–H groups of two mellitic acid molecules form an oxygen bond between the two molecules. The mellitic acid molecules could then form an anhydrous mellitic polymer. Stalport et al.^69^ finally conclude that both intra- and intermolecular reactions can occur. In this work, we carried out a computational spectroscopy simulation of the final expected photoproduct, i.e. benzenehexacarboxylic acid-trianhydride, and the intermediate photoproduct obtained by the loss of only one water molecule as shown in the third step of the pathway described in Fig. 7, and we compared their vibrational frequencies with the new photoproduct bands appeared during irradiation of mellitic acid. Since we irradiated only for 3 h compared to the 100 h of irradiation carried out by Stalport et al.^69^, we expected to have formed mainly the intermediate photoproduct and, indeed, we found the best matches with its vibrational modes. The new band at $1959 \, {\text{cm}}^{-1}$ can be tentatively assigned to the fundamental C$=$O stretching of the new anhydrous functional group, with a discrepancy of ${+60\text{ cm}}^{-1}$. The $1383 \, {\text{cm}}^{-1}$ band can be attributed to the fundamental COOH stretching-bending (mainly) and ring C–C stretching of the intermediate photoproduct with a discrepancy of ${-30\text{ cm}}^{-1}$. Finally, the $1261 \, {\text{cm}}^{-1}$ band can be assigned to the fundamental COOH bending of the intermediate photoproduct with a discrepancy of – 56 cm^−1^, but also gives a good match with a possible vibration of the final photoproduct (benzenehexacarboxylic acid-trianhydride) involving the ring C–C stretching and the three new anhydrous functional groups stretching and bending (discrepancy of – 8 cm^−1^). These discrepancies may be ascribed to the variations of the vibrational frequencies that the molecules can experience when they are embedded into a mineral matrix rather than the isolated molecules simulated in our computational study^70,71^. The new band at $3776 \, {\text{cm}}^{-1}$, instead, does not give a good match with either the final or the intermediate photoproduct, and may be ascribed to the anhydrous mellitic polymer resulting from possible intermolecular photoreactions, which would be quite likely in the pure solid state mellitic acid given the vicinity of the molecules in the structure.

For $10$ wt$\%$ mellitic acid adsorbed on hydrated magnesium sulfate, no significant degradation of molecular bands was observed. However, some of the bands that are indicative of formation of photoproducts were detected as in the case of pure mellitic acid, but with slower formation rates than the pure molecule. More precisely, four bands were formed: $1959 \, {\text{cm}}^{-1}$ ($5.1\,\upmu\text{m}$) (Fig. 6d), $1404 \, {\text{cm}}^{-1}$ $(7.1\,\upmu\text{m})$, $1383 \, {\text{cm}}^{-1}$ ($7.2\,\upmu\text{m}$) (Fig. 6f) and $1261 \, {\text{cm}}^{-1} \, \left(7.9\,\upmu\text{m}\right)$ (Fig. 6b). As shown in Table 4, for the $1959 \, {\text{cm}}^{-1}$ band, already observed in the case of UV irradiation of pure mellitic acid, we obtained $\alpha =(1.1\pm 0.1)\times {10}^{-3}{\text{ s}}^{-1}$ rather than $\alpha =(8\pm 3)\times {10}^{-3}{\text{ s}}^{-1}$ obtained in the case of pure mellitic acid. The new band at $1404 \, {\text{cm}}^{-1}$, not observed in the case of pure mellitic acid, attributable to fundamental C-COOH stretching and COOH stretching of the intermediate photoproduct with a discrepancy of $-9 \, {\text{cm}}^{-1}$ based on comparison with calculated vibrational frequencies, turns out not to be integrable due to its low intensity. For the $1383 \, {\text{cm}}^{-1}$ band, we obtained $\alpha =(5.5\pm 0.8)\times {10}^{-3}{\text{ s}}^{-1}$, while in the case of pure mellitic acid $\alpha =(1.0\pm 0.2)\times {10}^{-2}{\text{ s}}^{-1}$. Finally, for the photoproduct at $1261 \, {\text{cm}}^{-1}$, $\alpha =(7\pm 1)\times {10}^{-3}{\text{ s}}^{-1}$, while it is non-integrable in the pure mellitic case.

The slower rate of formation of the photoproduct derived from intramolecular photoreaction in the case of mellitic acid adsorbed on hydrated magnesium sulfate can be explained considering that part of the adsorbed molecules interact with magnesium sulfate through the carboxyl group, as inferred from the IR characterization, and the rotation of COOH carboxyl groups and the subsequent interaction between neighboring O–H groups producing anhydrous groups are inhibited for that part of the mellitic acid molecules. The intermolecular photoreaction also would be less likely when mellitic acid molecules are dispersed into the mineral matrix. The non-detection of a new band at $3776 \, {\text{cm}}^{-1}$ in the case of the mellitic acid adsorbed on magnesium sulfate further corroborates the assignment of this band to the anhydrous mellitic polymer that could form only in the case of the pure mellitic acid and not when mellitic acid is adsorbed on magnesium sulfate. Further proof of formation of the anhydrous mellitic polymer in the case of pure mellitic acid is that such a polymer is UV-resistant and can inhibit further photoreactions, resulting in a lower amount of photoproduct formed with respect to the case of mellitic acid adsorbed on magnesium sulfate, as observed in our experiments (Fig. 6).

### Implications for the NASA Mars 2020 mission

To explore the mineralogical and organic content of rocks, Perseverance abrades the surface and analyzes subsurface material using its proximity science instruments. For rover operational reasons, the abraded patches have so far been left exposed to the environmental conditions of the Martian surface for at least $1\text{ sol}$ before measurements are made with the proximity science instruments. Indeed, ground-in-the-loop imaging of the abraded patch is needed for assessment of proximity science instrument positioning after altering the target by abrasion. According to the simulated Jezero crater UV flux, the average half-lives estimated in this work are $\overline{{t}_{1/2}^{\text{COOH}}}=(0.7\pm 0.1)\text{ sol}$ and $\overline{{t}_{1/2}^{ring {\text{CH}}}}=(7.9\pm 0.8)\text{ sol}$ for the pure phthalic acid case (Table 1), while $\overline{{t}_{1/2}^{\text{COOH}}}=(1.2\pm 0.3)\text{ sol}$ for the pure mellitic acid one (Table 3). The half-lives estimated in this work suggest that the vibrational spectroscopic features of the carboxyl functional groups of phthalic and mellitic acid would likely be difficult to detect after $1\text{ sol}$ of exposure to Martian UV, while different photoproducts bands could be observed. However, if phthalic and mellitic acid are embedded into hydrated magnesium sulfate, all their vibrational spectroscopic features should still be detectable when the SHERLOC analysis is carried out, thanks to the photoprotective properties of magnesium sulfate, along with photoproduct bands diagnostic of the parent carboxylic acid molecules. Moreover, considering the total UV irradiation time reached in our experiments, we are confident that the invariance of their vibrational spectroscopic features is satisfied for at least $63$ Martian sols at Jezero crater when these organic molecules are embedded into hydrated magnesium sulfate. These results corroborate the hypothesis that the SHERLOC fluorescence signals observed in association with sulfates may be due to organics. Furthermore, the photoprotective properties of hydrated magnesium sulfate are consistent with the most intense SHERLOC fluorescence signals possibly due to organics being present in association with sulfates rather than other photocatalytic minerals that might cause degradation of organics earlier than SHERLOC analysis, sowing doubts on possible detection biases towards organics in sulfates and consequent underestimation of the astrobiological potential of the collected samples based on tardive SHERLOC analysis of abraded patches. Moreover, this work shows that the SuperCam IR, Raman, and time-resolved luminescence in-situ analysis may contribute to organics detection. In fact, some phthalic acid bands are still present in the SuperCam IR spectral range ($7700{-}3850 \, {\text{cm}}^{-1}$, $1.3{-}2.6\,\upmu\text{m}$) and Raman after its adsorption on hydrated magnesium sulfate, meaning that also SuperCam could potentially detect such organics in magnesium sulfate if present at wt$\%$ concentration (see Supplementary Table S5 for the specific wavelengths of the organic bands in the SuperCam spectral range). The mineral band shifts due to the presence of both these organics observed in Raman analysis might be further indirect evidence. These limitations of in situ investigations highlight the importance of Mars Sample Return (MSR) to characterize the actual organic content of the Martian samples collected by Perseverance. Therefore, analysis in terrestrial laboratories of sulfate samples will be crucial as we will be able to use more highly-targeted (higher magnification and long-working microscope objectives) and high-resolution spectroscopy (with beams that can reach $\sim 1{-}2\,\upmu\text{m}$ in diameter) to detect organics in the interiors of crystals (in fluid inclusions and as solid inclusions).

## Conclusions

This work reports the IR and Raman characterization and UV irradiation of Martian analog samples obtained adsorbing two carboxylic acids, i.e. phthalic and mellitic acid, on magnesium sulfate in water followed by desiccation.

The vibrational spectroscopy characterization of these samples shows that most of the characteristic vibrational bands of the pure molecules are either not present, or strongly attenuated, or in the case of mellitic acid undergo shifts, when the molecules are adsorbed on the sulfate. The vibrational spectroscopic features of the sulfate also change as consequence of molecular adsorption, indicating overall a mineral dehydration. This highlights the importance of acquiring databases of infrared spectroscopic features for organo-mineral complexes to support detection of organics on Mars through IR spectroscopy. Regarding molecule–mineral interaction, IR characterization shows the invariance of the IR features of phthalic acid when adsorbed on magnesium sulfate with respect to the pure organic compound, suggesting that phthalic acid might have been included into the magnesium sulfate crystals precipitated during desiccation. Instead, part of the mellitic acid molecules seems to interact with the sulfate through the carboxyl groups as suggested by the split of the $1153 \, {\text{cm}}^{-1}$ ($8.7\,\upmu\text{m}$) band assigned to the fundamental COO-H in-plane bending vibration.

UV irradiation experiments allowed us to assess the stability of these carboxylic acids, both pure and adsorbed on magnesium sulfate once exposed to the ambient Martian UV. Specifically, according to the average Martian UV flux value, the first effect of irradiation of pure phthalic acid is the degradation of the COOH carboxyl group with a weighted average half-life of $\overline{{t}_{1/2}^{\text{COOH}}}=(0.49\pm 0.06)\text{ sol}$, followed by the degradation of the ring C–H bonds with a weighted average half-life of $\overline{{t}_{1/2}^{ring {\text{CH}}}}=(6.5\pm 0.7)\text{ sol}$. The aromatic ring does not degrade, rather the intensity of the bands of some of its vibrational modes increases, suggesting an increase of aromaticity of the molecule as a consequence of UV irradiation. In addition, two new bands at $1383 \, {\text{cm}}^{-1} \left(7.2\,\upmu\text{m}\right)$ and $1261 \, {\text{cm}}^{-1}\, (7.9\,\upmu\text{m})$ appears during UV irradiation of pure phthalic acid, which are indicative of formation of photoproducts. For pure mellitic acid, degradation is observed only for the carboxyl group, more specifically for the bands related to the COO-H bond with a weighted average half-life of $\overline{{t}_{1/2}^{\text{COOH}}}=(1.0\pm 0.2)\text{ sol}$, and 4 bands appear at $3776 \, {\text{cm}}^{-1}$ ($2.7\,\upmu\text{m}$), $1959 \, {\text{cm}}^{-1}$ ($5.1\,\upmu\text{m}$), $1383 \, {\text{cm}}^{-1}$ ($7.2\,\upmu\text{m}$) and ${1261\text{ cm}}^{-1}$ ($7.9\,\upmu\text{m}$), due to the formation of photoproducts through intra- and inter-molecular photoreactions involving the carboxyl groups. In contrast, greater photostability is observed for both carboxylic acids when adsorbed on hydrated magnesium sulfate. Indeed, either no degradations or signals of change in the adjacent continuum not associated with band degradation are observed for the entire duration of our UV irradiation, corresponding to UV exposures for $48$ sols on Mars, while new bands indicative of photoproducts appear, as in the case of pure molecules, but with a slower formation rate. See the “Implications for the NASA Mars 2020 mission” section above for the half-life values scaled to the UV flux of the Jezero crater. These results indicate in general a photoprotective behavior of hydrated magnesium sulfate, despite the different modes of interaction between the two carboxylic acids and the mineral, which corroborates the hypothesis that sulfates might have played a key role in the preservation of organics on Mars, and that the fluorescence signals detected by SHERLOC in association with sulfates could potentially arise from organic compounds. Additionally, the photoprotective properties of sulfates may explain why the strongest SHERLOC fluorescence signals have been observed co-located with sulfates rather than other minerals with photocatalytic properties that may degrade organics prior to SHERLOC analysis. This raises concerns about potential biases in detecting organics within sulfates and the consequent potential underestimation of the astrobiological significance of collected samples based on delayed SHERLOC analysis of abraded patches. These limitations in on-site investigations underscore the necessity of Mars Sample Return (MSR) to accurately assess the organic content of Martian samples gathered by Perseverance.

Beyond the Mars 2020 mission, these studies are relevant also to other Martian rover exploration missions such as the future ExoMars/Rosalind Franklin mission and orbital remote sensing observations. In addition, since sulfates are widespread also on other rocky bodies in the Solar System such as the icy moons of gas giants, this laboratory analog work can support the future Europa Clipper mission as well as the interpretation of the data acquired by MIRI instrument of the James Webb Telescope (JWST).

## Methods

### Mars analog sample preparation

The Mars analog samples were prepared using epsomite (MgSO_4_-$7$H_2_O) as mineral phase ($>99.5\%$ purity, Sigma Aldrich), and two carboxylic acids: mellitic acid ($99\%$ purity, Sigma Aldrich) and phthalic acid $(>99.5\%$ purity, Sigma Aldrich).

To favor the establishment of physico-chemical interactions between the organic molecules and the mineral as in natural processes possibly occurred in aqueous early Martian environments, Mars analog samples were prepared suspending the mineral powder in an aqueous solution of the carboxylic acids and keeping the suspension under agitation on a test tube rotator for $24$ h, following a procedure described in Fornaro et al.^1^. Specifically, a mineral concentration of $140\text{ g}/{\text{L}}$ and $10$ wt% organic concentration was used. Such organic concentration is much higher than that expected on Mars based on Curiosity findings, but was chosen in order to be able to detect intense molecular IR bands and follow more easily the degradation kinetics during UV experiments. Finally, the suspensions were dried in oven in mild conditions (40 °C) to simulate a desiccation event possibly occurred on Mars in the past. For comparison, the epsomite blank was also prepared using the same procedure but without the organics.

### Infrared (IR) and Raman characterization

The characterization of the samples was performed by DRIFTS—Diffuse Reflectance Infrared Fourier Transform Spectroscopy and Raman spectroscopy. DRIFTS measurements were carried out at INAF-Astrophysical Observatory of Arcetri using a Bruker VERTEX 70v FTIR instrument equipped with a Praying Mantis™ Diffuse Reflection Accessory (Harrick DRIFT), using a Globar source, DigiTech DLaTGS detector, KBr beamsplitter. Spectra were acquired using $100$ scans of the interferometer with a resolution of $4 \, {\text{cm}}^{-1}$ in the wavenumber range $8000{-}400 \, {\text{cm}}^{-1}$ $(1.25{-}25\,\upmu\text{m})$. The Praying Mantis™ was saturated with nitrogen to reduce atmospheric contamination during the IR measurements, and to prevent oxidation during irradiation experiments. For Raman characterization, a Portable innoRam™ BWS445-532S (B&W TEKINC, Newark, USA) was used. The wavelength of the excitation laser implemented was of $532\text{ nm}$ ($45\text{ mW}$ laser output power) and the Raman signals were also collected by a CCD detector refrigerated by the Peltier effect. The spectral range of the Raman instrument was $65{-}3750 \, {\text{cm}}^{-1}$ with an average spectral resolution of $5 \, {\text{cm}}^{-1}$ (measured at $609\text{ nm}$). The spectral acquisition was performed using the BWSpec™ v.4.0215 (B&WTEKINC., Newark, USA).

### Ultraviolet (UV) irradiation experiments

The experimental setup for the UV irradiation consists of a Newport Oriel 300W Xenon discharge lamp (spectral range $200{-}930\text{ nm}$) interfaced with the Bruker VERTEX 70v interferometer, whose light is focused directly on the sample through an $800\,\upmu\text{m}$ optical fiber inserted into the sample chamber of the interferometer. With this configuration, the irradiated spot of the sample has an area of $7.07 \, {\text{mm}}^{2}$ and the UV lamp flux focused on the sample is ${\Phi }_{Lamp}=2.75\times {10}^{17}\text{ photons}\cdot {\text{s}}^{-1}\cdot {\text{cm}}^{-2}$ in the spectral range $200-400\text{ nm}$, measured through a Spectro 320 monochromator scanning spectrometer (Instrument System). Infrared spectra were recorded at regular intervals during UV irradiation for a total time of 10,800 s ($3$ h) to monitor the photodegradation. Specifically, infrared spectra were recorded initially every 5 s to monitor the quickest changes usually happening at the beginning of UV irradiation. Then, the time intervals between infrared measurements increased up to tens of minutes. The total degradation time was the same ($\text{10,800}$ s, 3 h) both for pure carboxylic acids and the carboxylic acids adsorbed on magnesium sulfate. This procedure allowed us to follow the degradation process in real time and the possible formation of new species by observing changes in the infrared spectroscopic characteristics^44^. The degradations of the same molecular bands in the case of the pure molecule and when the molecules are adsorbed on the mineral were compared to investigate the photoprotective/photocatalytic properties of the mineral. The relative areas $A$ of the same molecular bands for pure molecule and molecule adsorbed on magnesium sulfate were calculated for each spectrum using MATLAB R2022a software (MATLAB Version: 9.12.0.1975300 Update 3). The ratio $A(t)/A(0)$ was plotted versus the irradiation time $t$, where $A(t)$ is the area of the band at a given irradiation time $t$, proportional to the number of molecules at that time, and $A(0)$ is the area of the band before the irradiation process, proportional to the initial number of molecules at time $t=0$. A first-order kinetics function was used to fit the experimental data:

$$\frac{A(t)}{A(0)}=B{e}^{-\beta t}+C,$$

where $B$ is the fraction of molecules that interact with UV radiation, $\beta$ is the degradation rate and $C$ is the fraction of molecules that do not interact with UV radiation because of their position deep in the solid sample. UV radiation, in fact, can only penetrate to a depth of a few micrometers, as opposed to IR radiation. From the degradation rate $\beta$, obtained from the fit, it is possible to calculate the half-life ${t}_{1/2}$, or the time required to destroy $50\%$ of the initial number of molecules, using the following formula:

$${t}_\frac{1}{2}=\frac{\text{ln}2}{\beta }.$$

From $\beta$ it is also possible to calculate the UV destruction cross section $\sigma$ that represents the probability of interaction between UV radiation and molecule:

$$\sigma =\frac{\beta }{{\Phi }_{Lamp}},$$

where, ${\Phi }_{Lamp}$ is the total incident UV lamp flux mentioned above. In the case of formation of new peaks, the kinetics was investigated through the function:

$$A\left(t\right)=A\left(\infty \right)\left(1-{e}^{-\alpha t}\right),$$

where $A\left(t\right)$ is the area of the band at a given irradiation time $t$, $A(\infty )$ is the maximum value of the area at $t=\infty$ and $\alpha$ is the formation rate. Finally, to estimate the survivability of this molecule on Mars, half-lives were scaled on two different Martian UV flux values: the first one is the Patel et al.^5^ Martian UV flux in the $190-325\text{ nm}$ spectral region, assuming dust free atmosphere at the noontime equator, that is ${\Phi }_{Mars}^{Patel}=1.4\times {10}^{15}\text{ photons}\cdot {\text{s}}^{-1}\cdot {\text{cm}}^{-2}$, good for a general purpose evaluation; the second one is the Jezero Crater UV flux ${\Phi }_{Mars}^{Jezero}=1.06\times {10}^{15}\text{ photons}\cdot {\text{s}}^{-1}\cdot {\text{cm}}^{-2}$, more accurate for the Mars 2020 mission. The latter one is calculated for the same spectral region using the radiative transfer model COMIMART^72^, considering the right latitude ($18^\circ$ N), local noon, ${L}_{s}=0^\circ$ and including the state-of-the-art radiative properties of Martian dust with dust opacity $\tau =0.6$.

### Density functional theory (DFT) simulations

Analysis of experimental results and assignment of the InfraRed (IR) and Near InfraRed (NIR) spectra was supported by anharmonic computations, employing the Generalized second-order Vibrational Perturbation Theory approach (GVPT2)^73–76^, which allows for the direct simulation of IR spectra accounting for both energies and intensities for fundamental, overtones and combination bands. All quantities necessary for GVPT2 calculations have been computed by Density Functional Theory (DFT) employing the B3LYP functional^77^ with the double-zeta basis sets SNSD^74^, and including Grimme’s dispersion correction D3 (in conjunction with Becke-Johnson damping)^78–80^. This approach has been well tested for structural and spectroscopy properties of biological molecules of astrochemical interest^44,70,81–83^. In GVPT2 computations, the recommendations outlined in Bloino et al.^84^ have been followed. Moreover, the Large Amplitude-Motion (LAM) vibrations have been excluded from VPT2 by means of LAM-free VPT2 scheme^81^. Vibrations with harmonic frequencies below $100 \, {\text{cm}}^{-1}$ have been considered as LAMs. GVPT2 computations require appropriate definition of anharmonic resonances^85^. In this work it has been checked if all necessary Fermi resonances have been included in default settings, for mellitic acid and its Step1 photoproduct it was necessary to expand the resonances list, either directly or by lowering the threshold for the Minimum Difference PT2 vs Variational. All calculations were performed with Gaussian 16 suite of computer codes^86^ and using GaussView to visualize the normal modes and analyze in detail the outcome of vibrational computations and IR spectra. See Supplementary Table S6 for the complete computational results about phthalic acid, mellitic acid and its photoproducts. The molecular structures of the simulated compounds are shown in Supplementary Fig. S8.

### Supplementary Information

Supplementary Information.

## Data Availability

Data is provided within the manuscript or supplementary information files.
