# Supplementary material for: Investigating the stability of aromatic carboxylic acids in hydrated magnesium sulfate under UV irradiation to assist detection of organics on Mars
Source: Sci Rep. 2024 Jul 10;14:15945. doi: 10.1038/s41598-024-66669-8 (PMC11237158; doi:10.1038/s41598-024-66669-8)
Supplement: Supplementary file 1 — Supplementary Information. [file 41598_2024_66669_MOESM1_ESM.docx]

**Supplementary materials**

Investigating the stability of aromatic carboxylic acids in hydrated magnesium sulfate under UV irradiation to assist detection of organics on Mars

Andrew Alberini^1,2^ ([andrew.alberini@inaf.it](mailto:andrew.alberini@inaf.it)), Teresa Fornaro^1*^ ([teresa.fornaro@inaf.it](mailto:teresa.fornaro@inaf.it)), Cristina García Florentino^1,3^ (cristina.garcia.florentino@gmail.com), Malgorzata Biczysko^4^ (biczysko@i.shu.edu.cn), Iratxe Poblacion^3^ (iratxe.poblacion@ehu.eus), Julene Aramendia^3^ (julene.aramendia@ehu.eus), Juan Manuel Madariaga^3^ (juanmanuel.madariaga@ehu.eus), Giovanni Poggiali^1,5^ (giovanni.poggiali@inaf.it), Álvaro Vicente-Retortillo^6^ (adevicente@cab.inta-csic.es), Kathleen C. Benison^7^ ([kathleen.benison@mail.wvu.edu](mailto:kathleen.benison@mail.wvu.edu)), Sandra Siljeström^8^ (sandra.siljestrom@ri.se), Sole Biancalani^1,9,10,11^ ([sole.biancalani@inaf.it](mailto:sole.biancalani@inaf.it)), Christian Lorenz^1,12^ (christian.lorenz@unina.it), Edward A. Cloutis^13^ (e.cloutis@uwinnipeg.ca), Dan M. Applin^13^ (daniel.m.applin@gmail.com), Felipe Gómez^6^ (gomezgf@cab.inta-csic.es), Andrew Steele^14^ (asteele@carnegiescience.edu), Roger C. Wiens^15^ (rwiens@purdue.edu), Kevin P. Hand^16^ (kevin.p.hand@jpl.nasa.gov) & John R. Brucato^1^ (john.brucato@inaf.it)

*^1^ INAF- Astrophysical Observatory of Arcetri, L.go E. Fermi 5, 50125 Firenze, Italy*

*^2^ Department of Physics and Astronomy, University of Florence, Via Giovanni Sansone 1, 50019 Sesto Fiorentino, Florence*

*^3^ Department of Analytical Chemistry, University of the Basque Country UPV/EHU, 48080 Bilbao, Spain*

*^4^ College of Science, Shanghai University, 99 Shangda Road, Shanghai 200444, China*

*^5^ LESIA - Observatoire de Paris, Université Paris Cité, Université PSL, Sorbonne Université, CNRS, 5 place Jules Janssen, 92190 Meudon, France*

*^6^ Centro de Astrobiología (CAB), CSIC-INTA, Torrejón de Ardoz, Spain*

*^7^ Department of Geology and Geography, West Virginia University, Morgantown, WV, USA*

*^8^ RISE Research Institutes of Sweden, Stockholm, Sweden*

*^9^ Department of Physics, University of Trento, Via Sommarive 14, 38123 Povo*

*^10^ Italian Space Angency (ASI), viale del Politecnico snc, 00133, Rome, Italy*

*^11^Department of Earth Sciences, University of Florence, via G. La Pira 4, 50121, Florence, Italy*

*^12^ Department of Biology, University of Naples Federico II, Via Cinthia, 80126 Naples, Italy*

*^13^ Centre for Terrestrial and Planetary Exploration, University of Winnipeg, Winnipeg, Manitoba R3B 2E9, Canada*

*^14^ Carnegie Institute for Science, Washington, DC, USA*

*^15^ Earth, Atmospheric, and Planetary Sciences, Purdue University, West Lafayette, IN, USA*

*^16^ Jet Propulsion Laboratory, California Institute of Technology, Pasadena, CA, USA*

**Corresponding author*

**Table S1 – IR bands detectable both in pure phthalic acid spectrum and phthalic acid adsorbed on magnesium sulfate, with the vibrational mode assignment (in bold the main vibration) and intensity (w = weak; m = medium; s = strong), along with the wavenumber shifts with respect to the pure molecule.**

| Phthalic acid vibrational mode | Pure phthalic acid | | $\boldsymbol{10}$wt.% phthalic acid on magnesium sulfate | | |
| --- | --- | --- | --- | --- | --- |
|  | Wavenumber [cm^-1^] | Intensity | Wavenumber [cm^-1^] | Intensity | Shift [cm^-1^] |
| 1^st^ overtone ring C-H stretching** | **6050** | s | **6050** | m | 0 |
| 1^st^ overtone ring C-H stretching** | **6020** | m | **6018** | w | -2 |
| Combination COO-H stretching + ring C-H bending** | **5979** | w | **5977** | w | -2 |
| Combination ring C-H stretching + 1^st^ overtone ring C-H in-plane bending** | **5962** | m | **5960** | w | -2 |
| Combination ring C-H stretching + ring C-H in-plane bending** | **5836** | m | **5836** | w | 0 |
| 3^rd^ overtone ring C-C stretching and ring C-H in-plane bending** | **4787** | m | **4791** | w | +4 |
| Combination ring C-C stretching + ring C-H stretching* | **4677** | s | **4679** | m | +2 |
| Combination ring C-H stretching and bending + ring C-C stretching** | **4639** | m | **4639** | w | 0 |
| Combination ring C-H bending + ring C-H stretching* | **4582** | w | **4587** | w | +5 |
| Combination COO-H stretching and out-of-plane bending + ring C-H and ring C-C out-of-plane bending** | **4540** | w | **4540** | w | 0 |
| Combination C=O stretching + C=O bending** | **4505** | w | **4505** | w | 0 |
| 3^rd^ overtone ring C-H in-plane bending and ring C-C stretching** | **4457** | w | **4457** | w | 0 |
| Combination COO-H stretching and bending + ring C-C in-plane bending** | **4349** | m | **4349** | w | 0 |
| Combination COO-H bending + COO-H stretching* | **4156** | s | **4156** | w | 0 |
| Combination ring C-H and ring C-C in-plane bending and stretching + C-OOH stretching** | **4129** | m | **4127** | w | -2 |
| Combination ring C-H stretching and bending + C-OOH stretching** | **4085** | m | **4085** | w | 0 |
| Combination C=O stretching + COO-H in-plane bending + ring C-H in-plane bending** | **4046** | m | **4046** | w | 0 |
| Combination molecule torsion + COO-H stretching* | **3910** | s | **3910** | m | 0 |
| Combination COO-H stretching + COO-H bending and ring C-H bending + ring deformation** | **3746** | m | **3476** | w | 0 |
| Combination COO-H bending + ring C-C stretching* | **2905** | m | **2905** | w | 0 |
| Combination COO-H bending + COO-H stretching* | **2814** | w | **2814** | w | 0 |
| Fundamental ring C-H stretching and COO-H stretching* | **2658** | s | **2656** | s | -2 |
| 1^st^ overtone ring C-H in-plane bending** | **2529** | s | **2657** | s | -2 |
| Combination ring C-H in-plane bending + COOH bending** | **2423** | w | **2419** | w | -4 |
| Combination ring C-H in-plane and out-of-plane bending + COO-H bending** | **2253** | w | **2253** | w | 0 |
| Combination COO-H bending + ring C-C and C-H in-plane bending** | **2214** | s | **2214** | w | 0 |
| Combination ring C-H and C-C in-plane bending + C-OOH stretching** | **2099** | m | **2106** | w | +7 |
| Combination COO-H bending and ring C-H in-plane bending + ring C-H out-of-plane bending** | **2050** | m | **2054** | m | +4 |
| Combination ring C-C and C-H in-plane bending + whole molecule bending** | **2004** | m | **2006** | m | +2 |
| 1^st^ overtone ring C-H out-of-plane bending and ring C-C out-of-plane bending** | **1969** | s | **1969** | m | 0 |
| 1^st^ overtone ring C-H out-of-plane bending** | **1935** | m | **1931** | w | -4 |
| 3^rd^ overtone ring C-C and C-H in-plane bending and COOH bending** | **1896** | w | **1896** | w | 0 |
| Combination COO-H bending + ring C-H bending** | **1861** | m | **1859** | w | -2 |
| Fundamental C=O stretching and COO-H bending** | **1730** | s | **1726** | w | -4 |
| Fundamental C=O stretching* | **1693** | m | **1693** | w | 0 |
| Fundamental ring C-C stretching and ring C-H in-plane bending** | **1663** | m | **1659** | m | -4 |
| Fundamental ring C-C stretching* | **1589** | s | **1591** | m | +2 |
| Fundamental ring C-C stretching* | **1535** | m | **1537** | w | +2 |
| Fundamental ring C-C stretching* | **1499** | s | **1499** | m | 0 |
| Fundamental ring C-H in-plane bending* | **1454** | m | **1454** | m | 0 |
| Fundamental CO-O-H bending* | **1414** | s | **1410** | s | -4 |
| Fundamental ring C-C stretching* | **1314** | s | **1312** | s | -2 |
| Fundamental C-O stretching* | **1275** | m | **1275** | w | 0 |
| Fundamental ring C-H in-pane bending and ring C-C stretching (both carbons linked to COOH groups)** | **1261** | w | **1261** | w | 0 |
| Combination COOH + ring C-H and C-C out-of-plane bending** | **1213** | m | **1215** | w | +2 |
| Fundamental ring C-H bending* | **1155** | s | **1155** | w | 0 |
| Fundamental ring C-H in-plane bending* | **1142** | s | **1140** | w | -2 |
| Fundamental ring C-H in-plane bending* | **1111** | m | **1111** | w | 0 |
| Fundamental ring C-C in-plane bending* | **1074** | s | **1076** | w | +2 |
| 1^st^ overtone COO-H bending and ring C-H out-of-plane bending** | **1066** | w | **1066** | w | 0 |
| Fundamental ring C-H out-of-plane bending* | **1007** | s | **1007** | w | 0 |
| Fundamental ring C-H out-of-plane bending* | **974** | m | **974** | w | 0 |
| Fundamental ring C-C in-plane bending* | **831** | s | **831** | w | 0 |
| Fundamental ring C-H and C-C out-of-plane bending** | **800** | s | **800** | w | 0 |
| Fundamental ring C-H out-of-plane bending** | **787** | w | **789** | w | +2 |
| Whole molecule bonds bending** | **750** | w | **750** | w | 0 |
| Fundamental ring C-H out-of-plane bending* | **741** | s | **734** | w | -7 |
| Out-of-plane whole molecule bonds bending** | **694** | s | **696** | w | +2 |
| Fundamental ring C-C out-of-plane bending* | **679** | s | **683** | w | +4 |
| Fundamental COO-H bending** | **669** | w | **669** | w | 0 |
| Fundamental C=O and C-O in-plane bending* | **640** | s | **644** | w | +4 |
| Fundamental ring C-C in-plane bending* | **559** | s | **563** | w | +4 |

* ^1–5^

**** DFT calculations, this work**


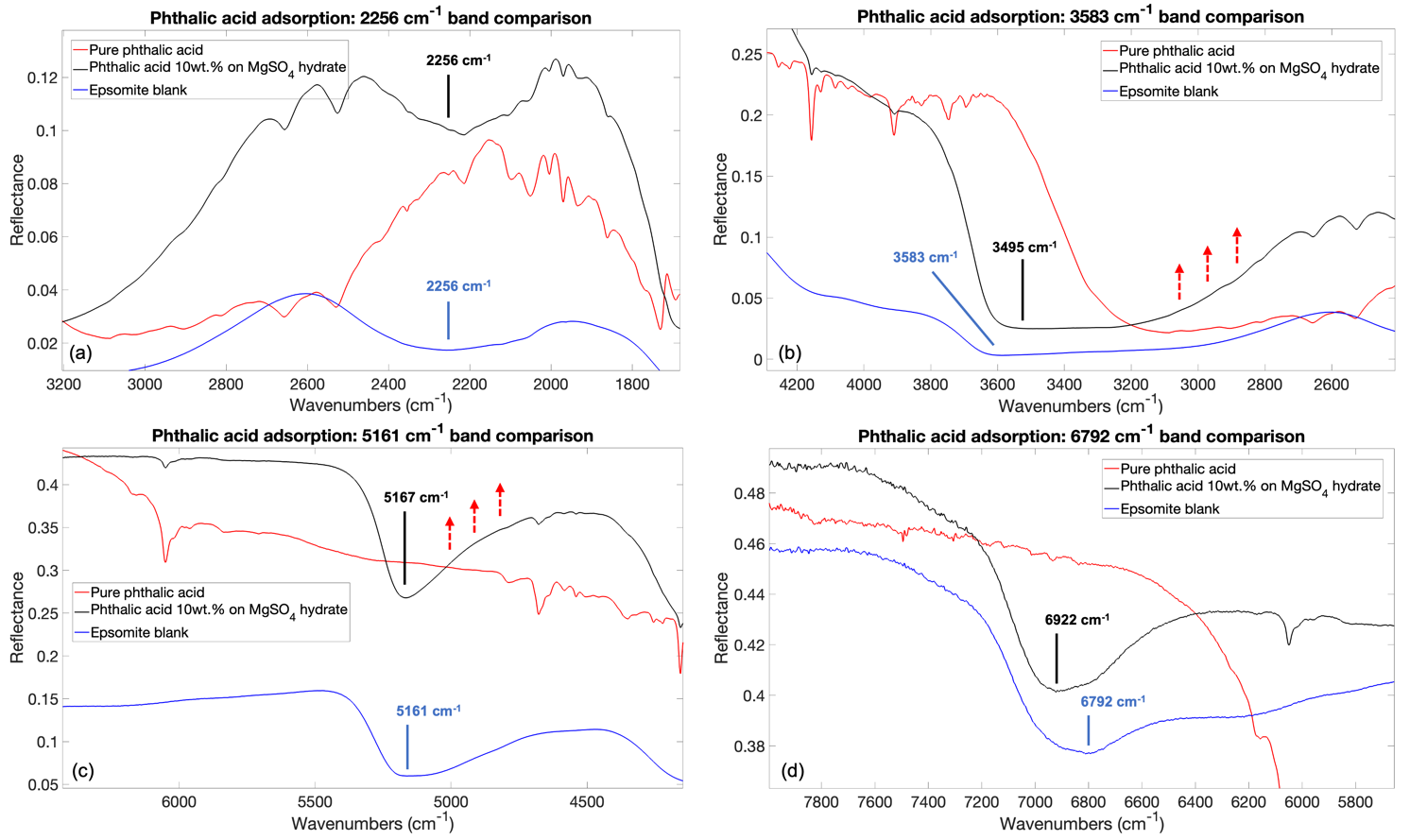


Figure S1: IR spectra comparison for pure phthalic acid, $10$ wt.$\%$ phthalic acid adsorbed on magnesium sulfate and epsomite blank regarding (a) hydrogen bonded water O-H stretching band at $2256$ ${cm}^{-1}$ ($4.4 \mu m$); (b) water O-H stretching band at $3583$ ${cm}^{-1}$ ($2.8 \mu m$); (c) $5163$ ${cm}^{-1}$ ($1.9 \mu m$) water O-H stretching and O-H bending combination band; (d) $6792$ ${cm}^{-1}$ ($1.5 \mu m$) $1$^st^ overtone of the water O-H stretching.

**Table S2 – IR bands detectable both in pure mellitic acid spectrum and mellitic acid adsorbed on magnesium sulfate, with the vibrational mode assignment (in bold the main vibration) and intensity (w = weak; m = medium; s = strong), along with the wavenumber shifts with respect to the pure molecule.**

| Mellitic acid vibrational mode | Pure mellitic acid | | $\boldsymbol{10}$wt.% mellitic acid on magnesium sulfate | | |
| --- | --- | --- | --- | --- | --- |
|  | Wavenumbers [cm^-1^] | Intensity | Wavenumbers [cm^-1^] | Intensity | Shift [cm^-1^] |
| Fundamental COO-H stretching* | **2667** | m | **2667** | w | 0 |
| Fundamental COO-H stretching* | **2555** | m | **2555** | w | 0 |
| Fundamental C=O stretching** | **1732** | m | **1730** | m | -2 |
| Fundamental C=O stretching** | **1715** | w | **1711** | w | -4 |
| Fundamental ring C-C stretching* | **1583** | s | **1585** | m | +2 |
| Fundamental ring C-C stretching** | **1574** | s | **1574** | m | 0 |
| Combination COO-H out-of-plane bending + ring C-C in-plane bending** | **1564** | s | **1564** | m | 0 |
| Fundamental COO-H bending** | **1474** | s | **1474** | s | 0 |
| Fundamental ring C-C stretching* | **1445** | m | **1445** | m | 0 |
| Fundamental COOH in-plane bending and ring breathing** | **1366** | s | **1366** | m | 0 |
| Fundamental COOH bending** | **1311** | w | **1311** | w | 0 |
| Fundamental COOH vibrations* | **1288** | s | **1288** | m | 0 |
| Fundamental COO-H bending** | **1199** | s | **1198** | m | -1 |
| Fundamental COO-H in-plane bending** | **1190** | m | **1190** | m | 0 |
| Fundamental COO-H in-plane bending and C-O stretching** | **1153** | s | **1151** | m | -2 |
| Fundamental carboxylic C-COOH bending** | **872** | w | **872** | w | 0 |
| Fundamental COO-H bending** | **858** | w | **858** | w | 0 |
| Combination COOH bending + ring C-C out-of-plane bending** | **845** | w | **845** | w | 0 |
| Fundamental COOH bending and ring C-C out-of-plane bending** | **795** | w | **793** | w | -2 |
| Fundamental COO-H bending** | **665** | w | **667** | w | +2 |
| Fundamental COO-H bending** | **596** | s | **600** | m | +4 |

***** ^6^

**** DFT calculations, this work**


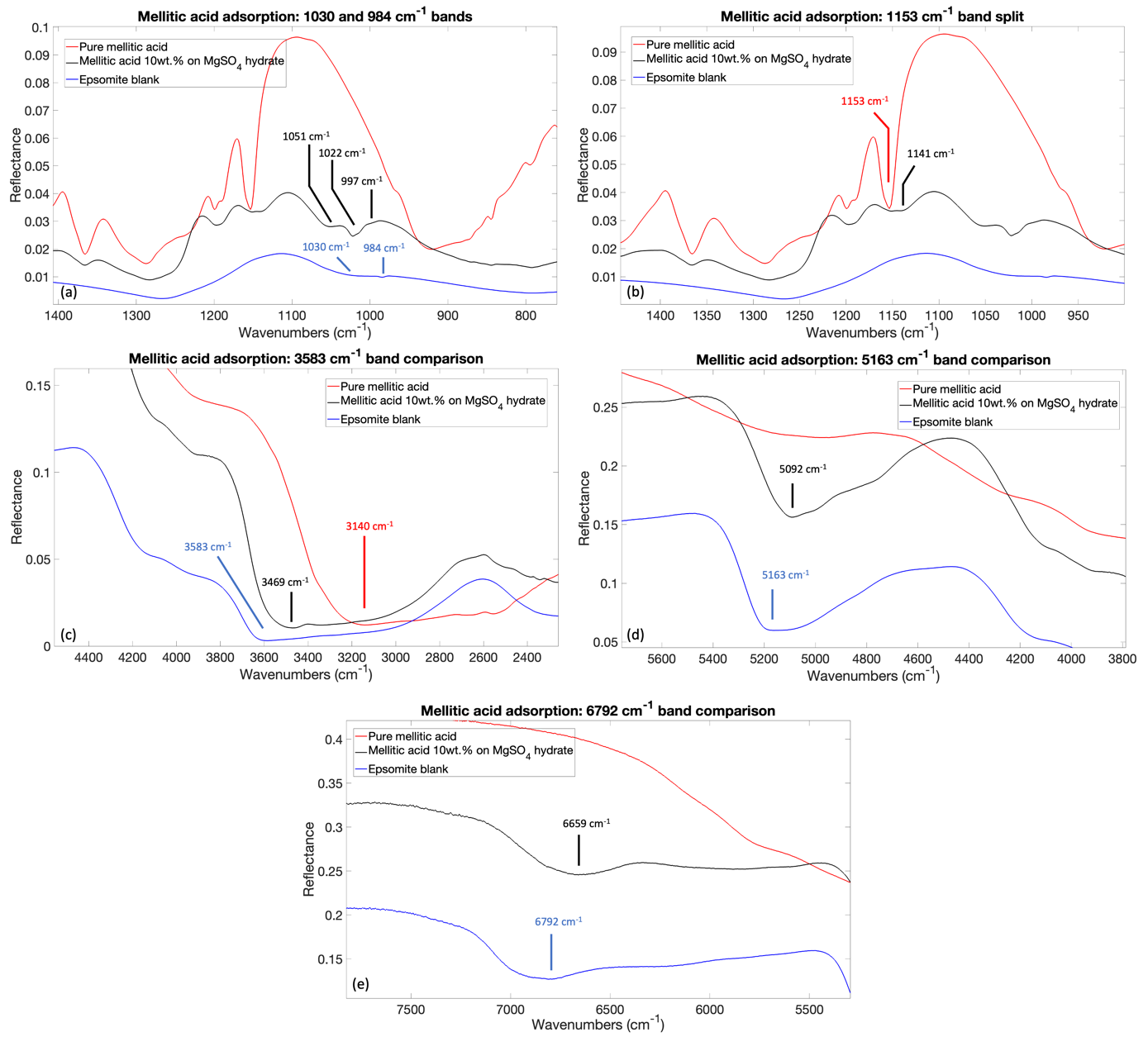


Figure S2: IR spectra comparison for pure mellitic acid, $10$ wt.$\%$ mellitic acid adsorbed on magnesium sulfate and epsomite blank regarding (a) symmetric sulfate vibration at $984$ ${cm}^{-1}$ ($10.2 \mu m$); (b) split (in post-adsorption) of the $1153$ ${cm}^{-1}$ ($8.7 \mu m$) band of pure mellitic acid associated computationally mainly with the fundamental COO-H in-plane bending vibration; (c) water O-H stretching at $3583$ ${cm}^{-1} (2.8 \mu m)$; (d) combination of water O-H stretching and O-H bending at $5163$ ${cm}^{-1} (1.9 \mu m)$; (e) 1^st^ overtone of the water O-H stretching at $6792$ ${cm}^{-1} (1.5 \mu m)$.

**Table S3 – Pure phthalic acid degradation results with A and C parameters.**

| Band [$\boldsymbol{cm}^{\boldsymbol{-1}}$] | Vibrational mode | $\boldsymbol{A}$ | $\boldsymbol{C}$ |
| --- | --- | --- | --- |
| 4046 | Combination **carboxylic C=O stretching** + carboxylic COO-H in-plane bending + ring C-H in-plane bending | $\left( 2.9\pm0.5 \right)\cdot{10}^{-2}$ | $\left( 9.7\pm0.1 \right)\cdot{10}^{-1}$ |
| 2423 | Combination ring C-H in-plane bending + carboxylic COOH bending | $\left( 3.3\pm0.6 \right)\cdot{10}^{-2}$ | $\left( 9.4\pm0.1 \right)\cdot{10}^{-1}$ |
| 1969 | 1° overtone **ring C-H out-of-plane bending** and ring C-C out-of-plane bending | $\left( 3.3\pm0.2 \right)\cdot{10}^{-2}$ | $\left( 9.6\pm0.1 \right)\cdot{10}^{-1}$ |
| 1693 | Fundamental carboxylic C=O stretching | $\left( 2.6\pm0.3 \right)\cdot{10}^{-2}$ | $\left( 9.7\pm0.1 \right)\cdot{10}^{-1}$ |
| 1275 | Fundamental carboxylic C-O stretching | $\left( 6.6\pm0.5 \right)\cdot{10}^{-2}$ | $\left( 9.3\pm0.1 \right)\cdot{10}^{-1}$ |
| 1213 | Combination carboxylic COOH bending + ring C-H and C-C out-of-plane bending | $\left( 2.0\pm0.2 \right)\cdot{10}^{-2}$ | $\left( 9.8\pm0.1 \right)\cdot{10}^{-1}$ |
| 1155 | Fundamental ring C-H bending | $\left( 1.3\pm0.3 \right)\cdot{10}^{-2}$ | $\left( 9.9\pm0.3 \right)\cdot{10}^{-1}$ |
| 1142 | Fundamental ring C-H in-plane bending | $\left( 2.9\pm0.3 \right)\cdot{10}^{-2}$ | $\left( 9.70\pm0.03 \right)\cdot{10}^{-1}$ |
| 1111 | Fundamental ring C-H in-plane bending | $\left( 2.2\pm0.4 \right)\cdot{10}^{-2}$ | $\left( 9.74\pm0.05 \right)\cdot{10}^{-1}$ |
| 1074 | Fundamental ring C-C in-plane bending | $\left( 1.12\pm0.09 \right)\cdot{10}^{-2}$ | $\left( 9.87\pm0.01 \right)\cdot{10}^{-1}$ |
| 1007 | Fundamental ring C-H out-of-plane bending | $\left( 1.1\pm0.1 \right)\cdot{10}^{-2}$ | $\left( 9.89\pm0.01 \right)\cdot{10}^{-1}$ |
| 974 | Fundamental ring C-H out-of-plane bending | $\left( 1.9\pm0.3 \right)\cdot{10}^{-2}$ | $\left( 9.83\pm0.03 \right)\cdot{10}^{-1}$ |
| 800 | Fundamental ring C-H and C-C out-of-plane bending | $\left( 1.3\pm0.2 \right)\cdot{10}^{-2}$ | $\left( 9.90\pm0.02 \right)\cdot{10}^{-1}$ |
| 741 | Fundamental ring C-H out-of-plane bending | $\left( 2.3\pm0.5 \right)\cdot{10}^{-2}$ | $\left( 9.73\pm0.05 \right)\cdot{10}^{-1}$ |


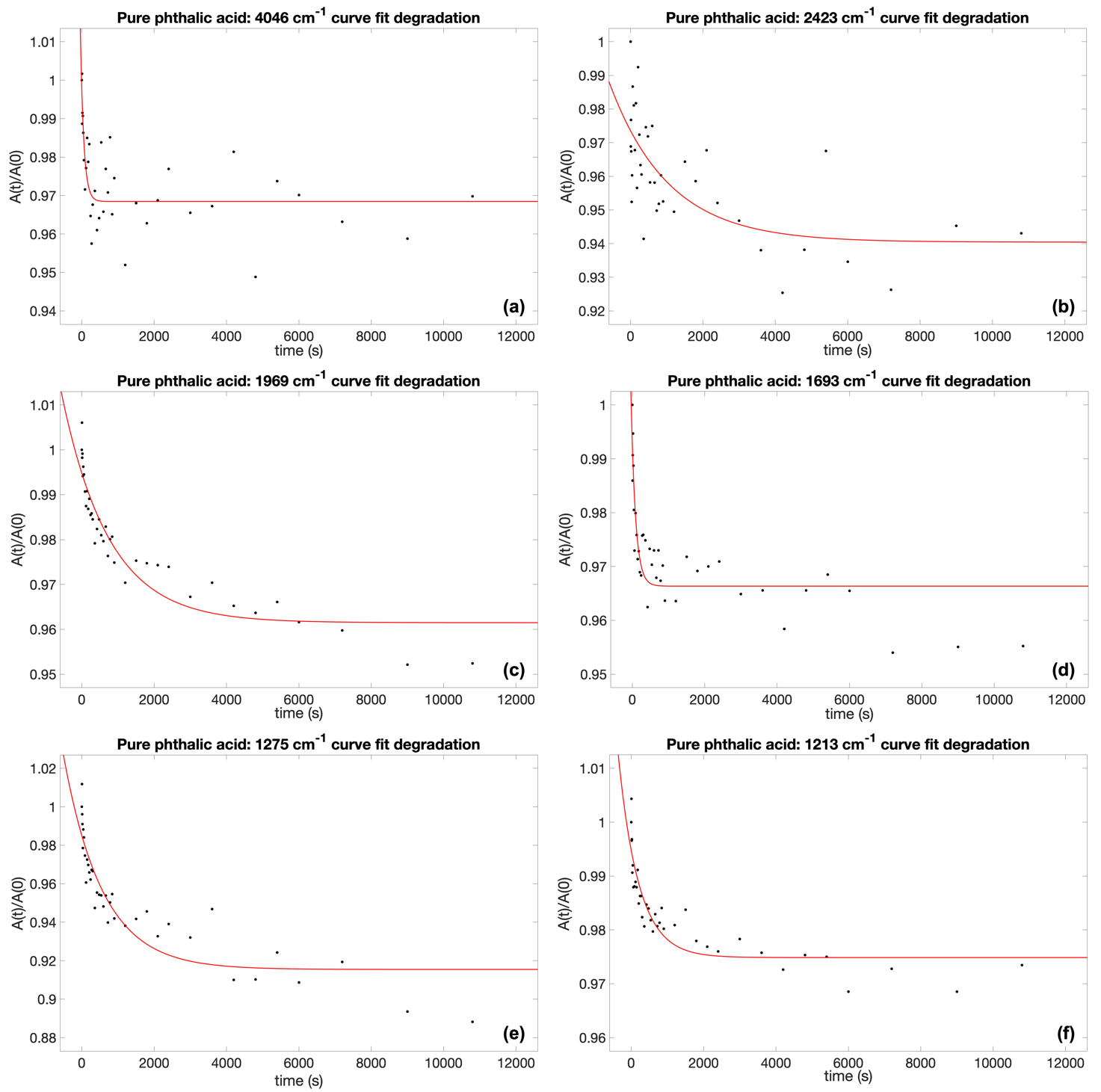


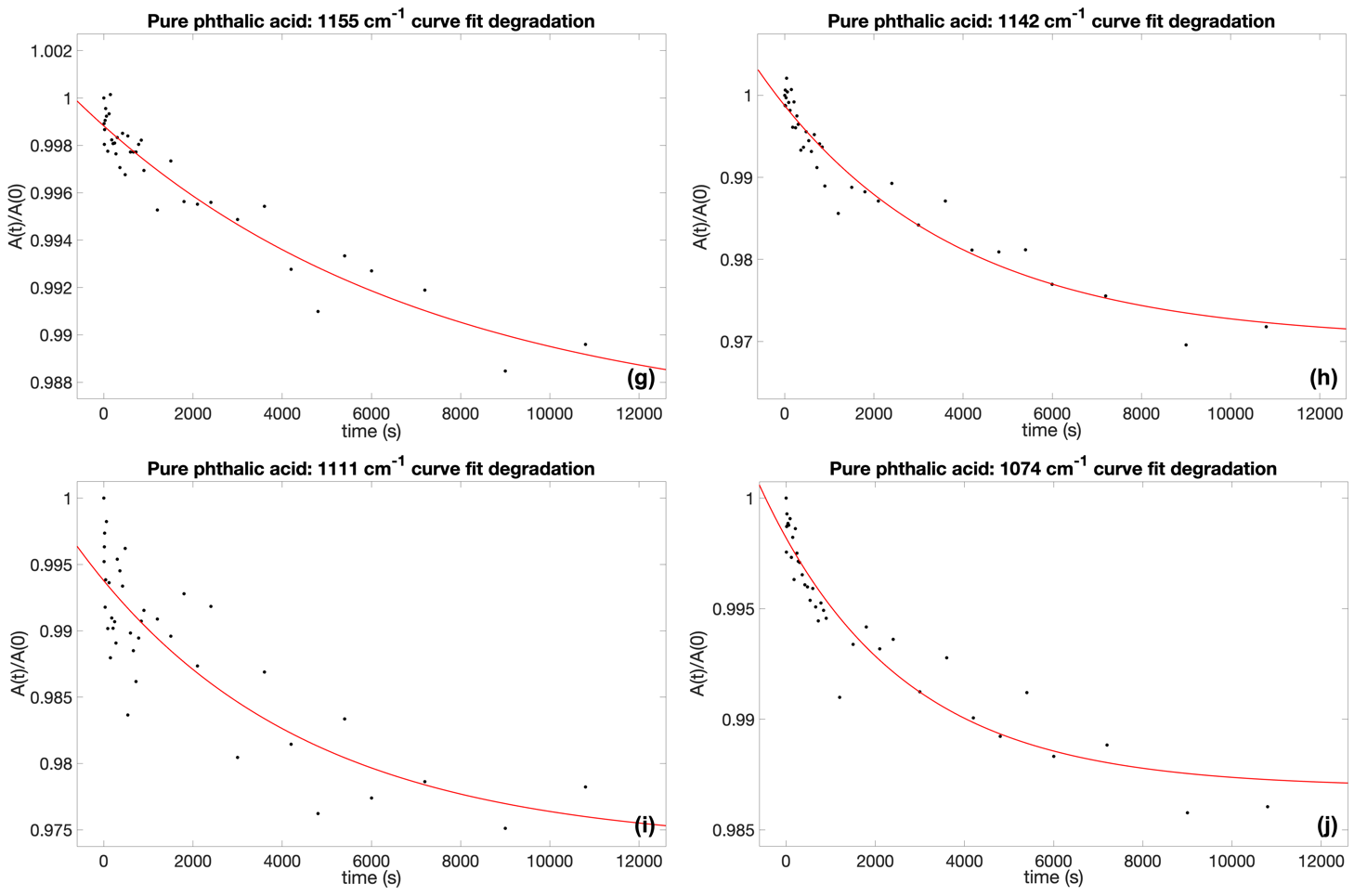


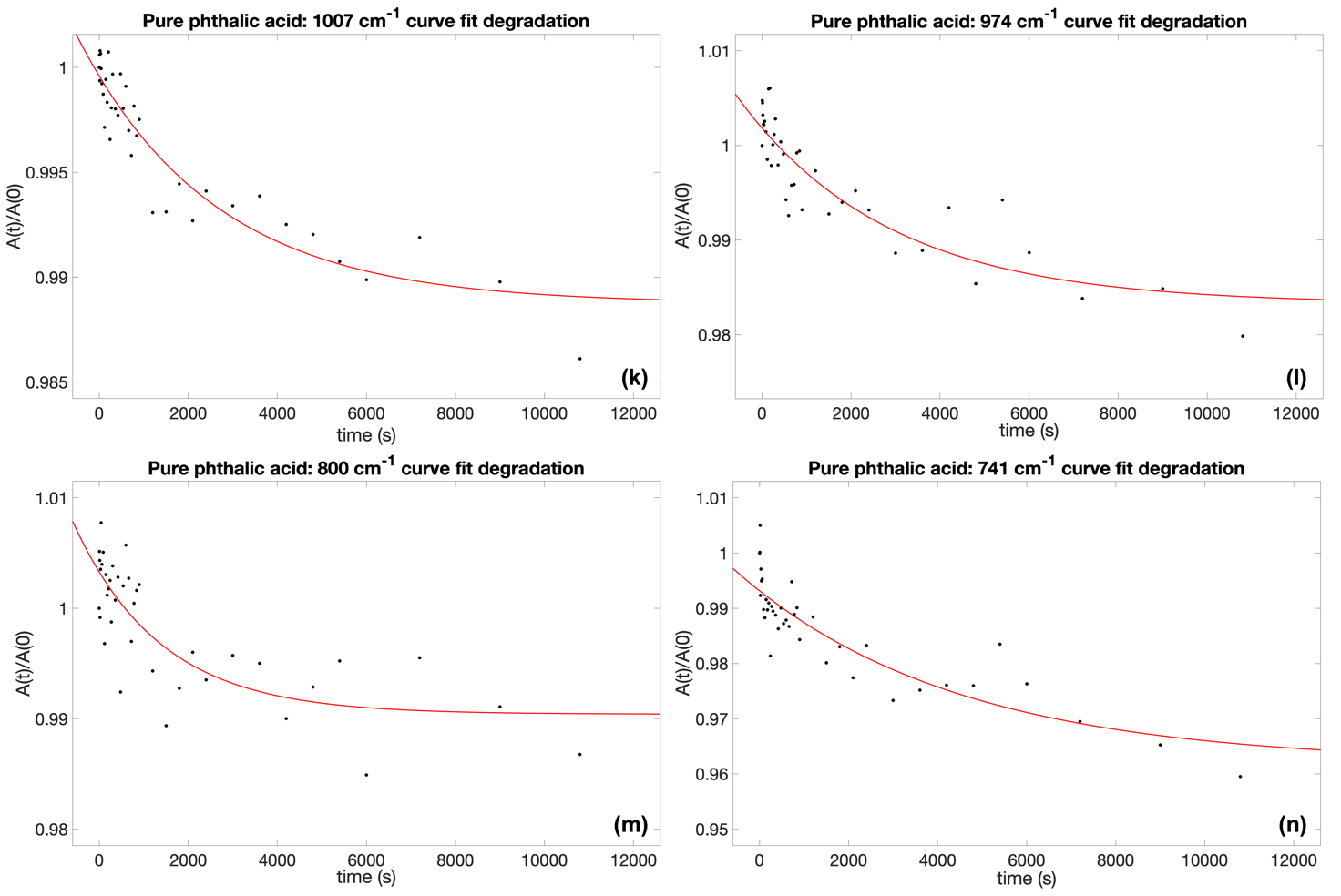


Figure S3: Curve fit degradation results for pure phthalic acid: (a) combination mainly assigned to carboxylic C=O stretching $4046 {cm}^{-1}$ band; (b) combination ring C-H in-plane bending + carboxylic COOH bending $2423 {cm}^{-1}$ band; (c) 1^st^ overtone mainly assigned to ring C-H out-of-plane bending $1969 {cm}^{-1}$ band; (d) fundamental carboxyl C$=$O stretching at $1693 {cm}^{-1}$ band; (e) fundamental carboxyl C-O stretching at $1275 {cm}^{-1}$ band; (f) combination carboxylic COOH bending + ring C-H and C-C out-of-plane bending $1213$ ${cm}^{-1}$ band; (g) fundamental ring C-H bending $1155$ ${cm}^{-1}$ band; (h) fundamental ring C-H in-plane bending $1142$ ${cm}^{-1}$ band; (i) fundamental ring C-H in-plane bending $1111$ ${cm}^{-1}$ band; (j) fundamental ring C-C in-plane bending $1074$ ${cm}^{-1}$ band; (k) fundamental ring C-H out-of-plane bending $1007$ ${cm}^{-1}$ band; (l) fundamental ring C-H out-of-plane bending at $974$ ${cm}^{-1}$ band; (m) fundamental ring C-H and C-C out-of-plane bending $800$ ${cm}^{-1}$ band; (n) fundamental ring C-H out-of-plane bending $741$ ${cm}^{-1}$ band.


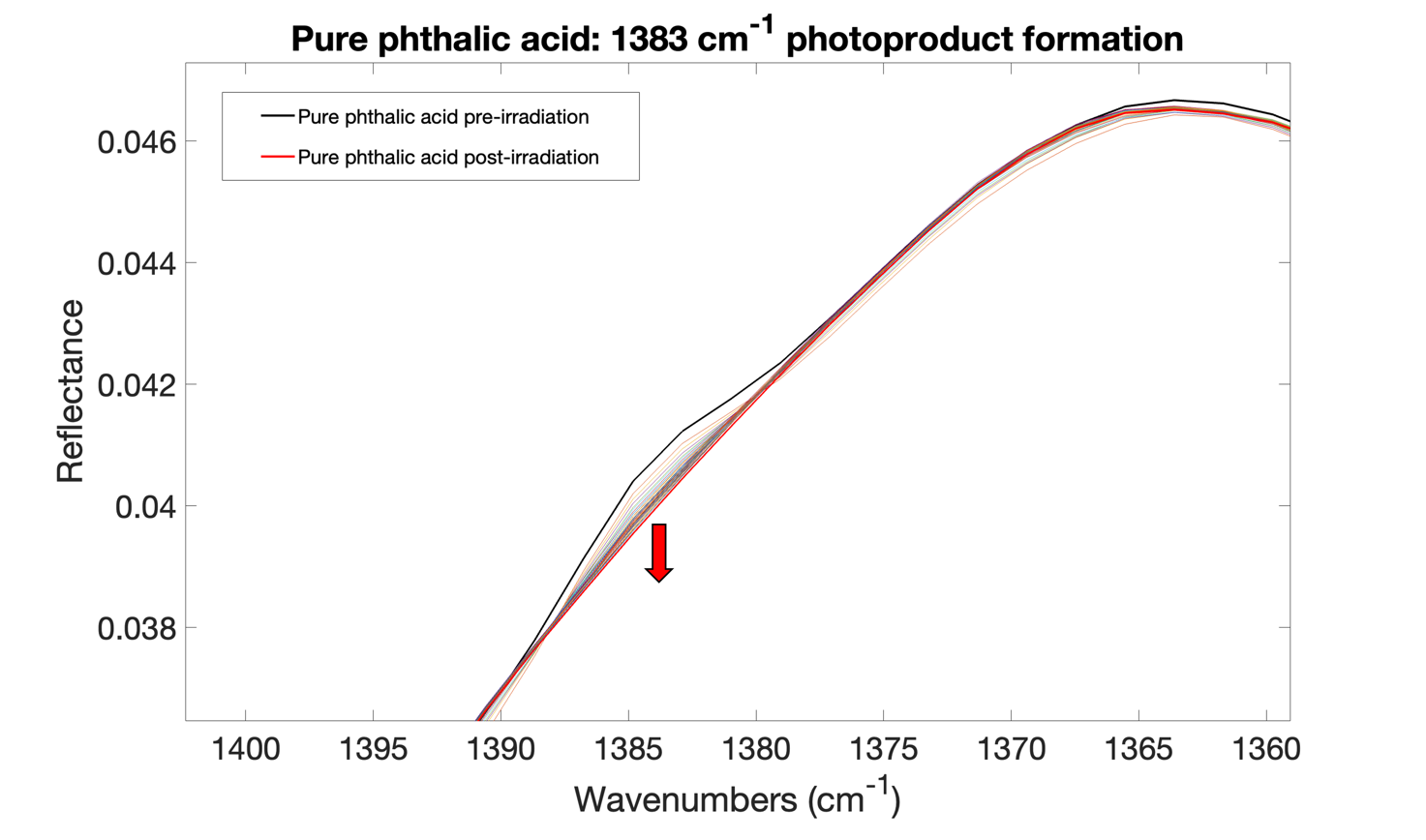


Figure S4: Pure phthalic acid photoproduct band at $1383 {cm}^{-1}$. IR spectra changing during irradiation experiment not integrable due to its low intensity.


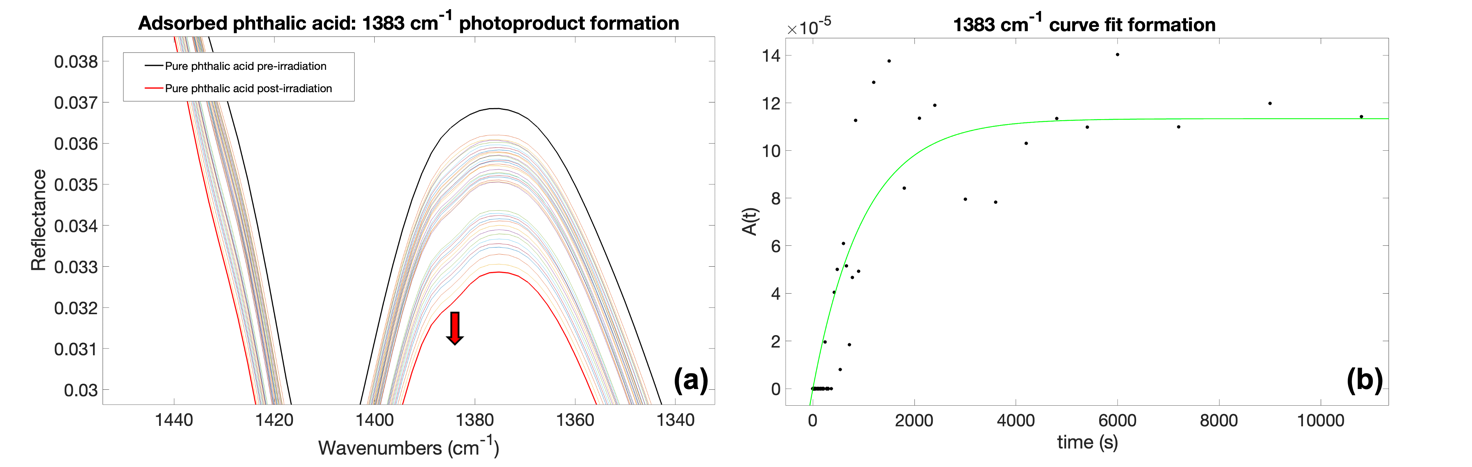


Figure S5: Photoproduct band at $1383 {cm}^{-1}$ appearing during UV irradiation of phthalic acid adsorbed on magnesium sulfate where (a) IR spectra changing during irradiation experiment; (b) curve fit formation with $\alpha=\left( 1.0\pm0.3 \right)\cdot{10}^{-3}s^{-1}$ and formation cross section of $\sigma_{f}=\left( 3.6\pm0.9 \right)\cdot{10}^{-21}{cm}^{2}$.

.


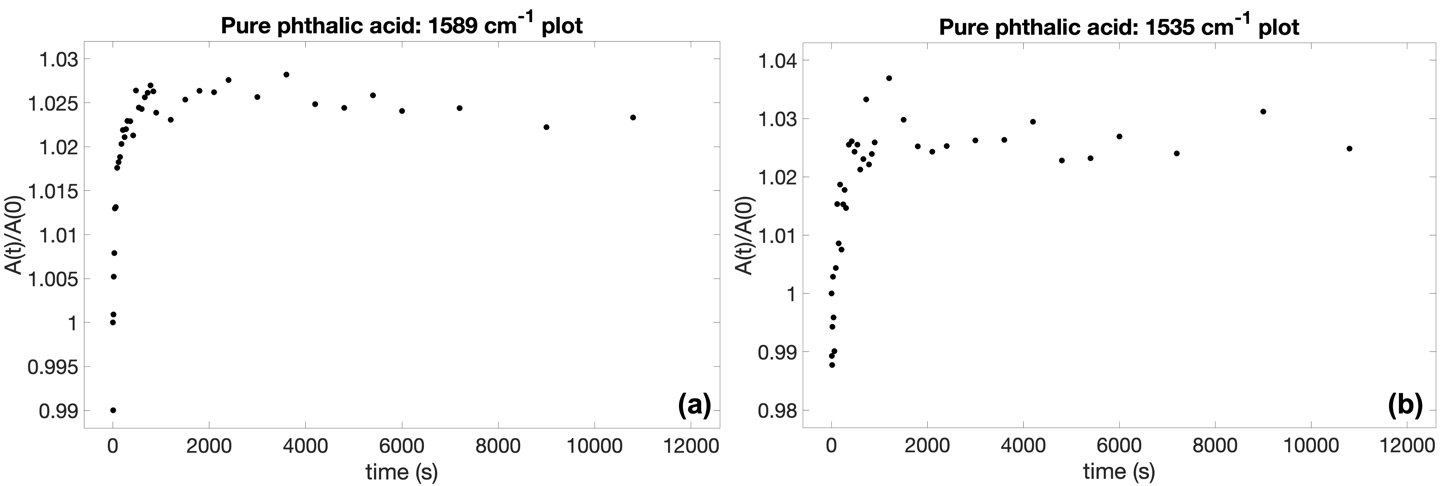


Figure S6: Pure phthalic acid ring C-C stretching increasing plots: (a) $1589$ ${cm}^{-1}$ band; (b) $1535 {cm}^{-1}$ band. Both not integrable with the fit model used.

**Table S4 – Pure mellitic acid degradation results with A and C parameters.**

| Band [$\boldsymbol{cm}^{\boldsymbol{-1}}$] | Vibrational mode | $\boldsymbol{A}$ | $\boldsymbol{C}$ |
| --- | --- | --- | --- |
| 1564 | Combination **carboxylic COO-H out-of-plane bending** + ring C-C in-plane bending | $\left( 1.3\pm0.1 \right)\cdot{10}^{-2}$ | $\left( 9.79\pm0.01 \right)\cdot{10}^{-1}$ |
| 1366 | Fundamental carboxylic COOH in-plane bending and ring ring breathing | $\left( 6\pm2 \right)\cdot{10}^{-3}$ | $\left( 9.89\pm0.02 \right)\cdot{10}^{-1}$ |
| 1153 | Fundamental **carboxylic COO-H in-plane bending** and C-O stretching | $\left( 6.2\pm0.4 \right)\cdot{10}^{-3}$ | $\left( 9.93\pm0.01 \right)\cdot{10}^{-1}$ |


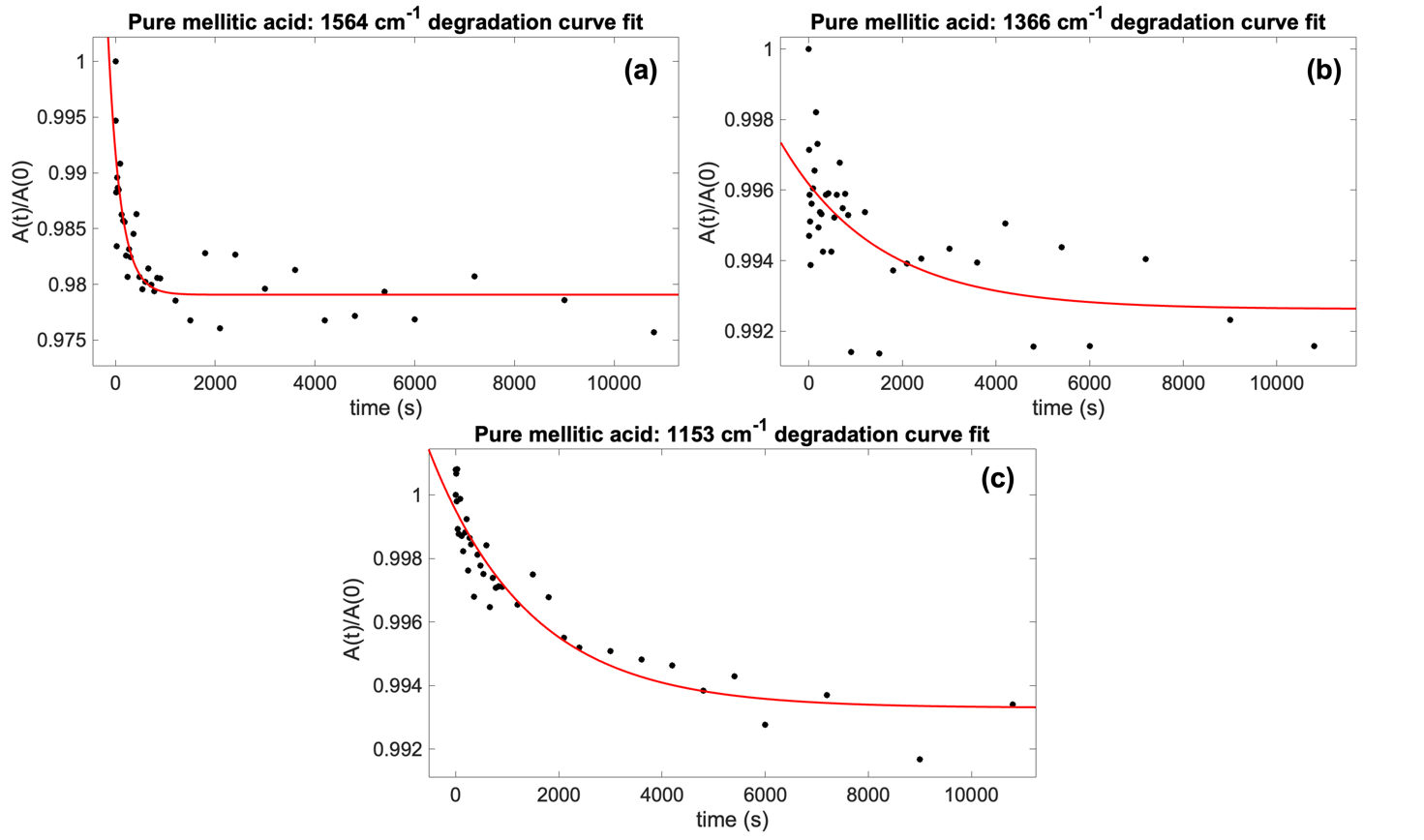


Figure S7: Curve fit degradation results for pure mellitic acid: (a) combination mainly assigned to carboxylic COO-H out-of-plane bending $1564 {cm}^{-1}$ band; (b) fundamental carboxylic COOH in-plane bending and ring breathing $1366 {cm}^{-1}$ band; (c) fundamental mainly assigned to carboxylic COO-H in-plane bending $1153 {cm}^{-1}$ band.


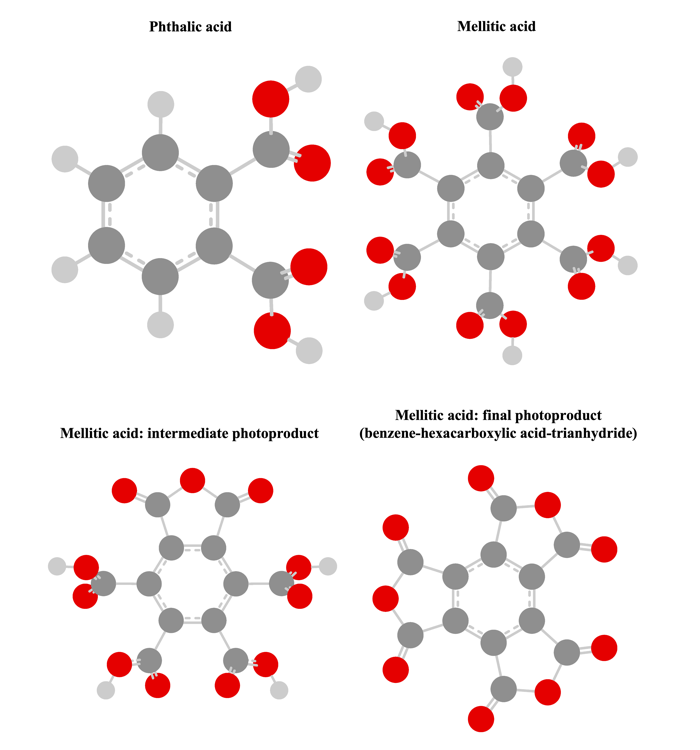


Figure S8: Structures of phthalic acid, mellitic acid, intermediate and final photoproduct (benzene-hexacarboxylic acid-trianhydride) of mellitic acid, optimized at the B3LYP-D3/SNSD level of theory.

**Table S5 – Bands of phthalic acid when adsorbed on magnesium sulfate in the IR SuperCam spectral range (**$\boldsymbol{7700-3850}$ $\boldsymbol{cm}^{\boldsymbol{-1}}$**,** $\boldsymbol{1.3-2.6}$ $\boldsymbol{\mu m}$**) with the vibrational mode assignment (in bold the main vibration) and intensity (w = weak; m = medium).**

| Phthalic acid vibrational mode | Wavenumber [cm^-1^] | Wavelength [$\boldsymbol{\mu m}$] | Intensity |
| --- | --- | --- | --- |
| 1^st^ overtone ring C-H stretching** | **6050** | **1.65** | m |
| 1^st^ overtone ring C-H stretching** | **6018** | **1.66** | w |
| Combination COO-H stretching + ring C-H bending** | **5977** | **1.67** | w |
| Combination ring C-H stretching + 1^st^ overtone ring C-H in-plane bending** | **5960** | **1.68** | w |
| Combination ring C-H stretching + ring C-H in-plane bending** | **5836** | **1.71** | w |
| 3^rd^ overtone ring C-C stretching and ring C-H in-plane bending** | **4791** | **2.09** | w |
| Combination ring C-C stretching + ring C-H stretching* | **4679** | **2.14** | m |
| Combination ring C-H stretching and bending + ring C-C stretching** | **4639** | **2.16** | w |
| Combination ring C-H bending + ring C-H stretching* | **4587** | **2.18** | w |
| Combination COO-H stretching and out-of-plane bending + ring C-H and ring C-C out-of-plane bending** | **4540** | **2.20** | w |
| Combination C=O stretching + C=O bending** | **4505** | **2.22** | w |
| 3^rd^ overtone ring C-H in-plane bending and ring C-C stretching** | **4457** | **2.24** | w |
| Combination COO-H stretching and bending + ring C-C in-plane bending** | **4349** | **2.30** | w |
| Combination COO-H bending + COO-H stretching* | **4156** | **2.41** | w |
| Combination ring C-H and ring C-C in-plane bending and stretching + C-OOH stretching** | **4127** | **2.42** | w |
| Combination ring C-H stretching and bending + C-OOH stretching** | **4085** | **2.45** | w |
| Combination C=O stretching + COO-H in-plane bending + ring C-H in-plane bending** | **4046** | **2.47** | w |
| Combination molecule torsion + COO-H stretching* | **3910** | **2.56** | m |

* ^1–5^

**** DFT calculations, this work**

**Table S6 – Vibrational wavenumbers and intensities calculated at anharmonic level using GVPT2 method and B3LYP-D3/SNSD level of theory for: phthalic acid, mellitic acid, intermediate photoproduct of mellitic acid, final mellitic photoproduct (benzene-hexacarboxylic acid-trianhydride). Legend: Mode (n) vibrational modes, E (harm) is the** $\boldsymbol{cm}^{\boldsymbol{-1}}$ **harmonic wavenumber, E (anharm) is the** $\boldsymbol{cm}^{\boldsymbol{-1}}$ **anharmonic wavenumber, I (harm) is the harmonic intensity in** $\boldsymbol{km/mol}$**, I (anharm) is the anharmonic intensity in** $\boldsymbol{km/mol}$**.**

**Phthalic acid fundamentals**

| Mode (n) | E (harm) | E (anharm) | I (harm) | I (anharm) |
| --- | --- | --- | --- | --- |
| 1(1) | 3748,15 | 3560,32 | 94,48 | 69,99 |
| 2(1) | 3747,68 | 3559,68 | 82,72 | 67,45 |
| 3(1) | 3210,24 | 3072,28 | 4,55 | 4,51 |
| 4(1) | 3207,01 | 3050,91 | 2,81 | 2,17 |
| 5(1) | 3193,60 | 3062,35 | 7,31 | 9,44 |
| 6(1) | 3179,74 | 3063,62 | 1,40 | 1,72 |
| 7(1) | 1812,83 | 1776,75 | 328,32 | 153,06 |
| 8(1) | 1793,49 | 1761,67 | 296,12 | 92,09 |
| 9(1) | 1640,97 | 1602,09 | 9,09 | 1,46 |
| 10(1) | 1623,98 | 1586,79 | 11,47 | 7,58 |
| 11(1) | 1526,72 | 1487,94 | 1,47 | 0,01 |
| 12(1) | 1475,48 | 1446,16 | 1,24 | 0,76 |
| 13(1) | 1370,76 | 1342,08 | 69,30 | 10,77 |
| 14(1) | 1367,35 | 1345,89 | 134,09 | 15,63 |
| 15(1) | 1351,07 | 1321,46 | 0,50 | 7,79 |
| 16(1) | 1300,83 | 1279,59 | 7,38 | 0,47 |
| 17(1) | 1218,96 | 1185,92 | 46,26 | 13,55 |
| 18(1) | 1209,35 | 1172,70 | 356,97 | 174,26 |
| 19(1) | 1187,12 | 1174,37 | 0,08 | 0,04 |
| 20(1) | 1158,11 | 1138,61 | 100,37 | 49,00 |
| 21(1) | 1122,22 | 1092,36 | 25,19 | 15,09 |
| 22(1) | 1066,93 | 1049,58 | 163,14 | 163,21 |
| 23(1) | 1063,62 | 1047,87 | 1,57 | 0,90 |
| 24(1) | 1012,86 | 986,36 | 0,68 | 0,62 |
| 25(1) | 982,74 | 960,52 | 5,23 | 5,81 |
| 26(1) | 908,36 | 892,64 | 0,19 | 0,05 |
| 27(1) | 811,98 | 797,37 | 19,73 | 17,64 |
| 28(1) | 811,85 | 802,15 | 4,68 | 2,26 |
| 29(1) | 803,82 | 796,27 | 8,65 | 3,29 |
| 30(1) | 752,34 | 739,14 | 21,67 | 13,34 |
| 31(1) | 751,19 | 728,84 | 120,57 | 31,53 |
| 32(1) | 712,93 | 699,70 | 0,61 | 0,74 |
| 33(1) | 637,57 | 631,24 | 24,36 | 19,70 |
| 34(1) | 636,03 | 629,43 | 61,22 | 63,90 |
| 35(1) | 605,37 | 582,93 | 105,53 | 107,41 |
| 36(1) | 600,94 | 586,00 | 20,26 | 12,69 |
| 37(1) | 565,77 | 556,97 | 17,90 | 14,47 |
| 38(1) | 547,88 | 534,83 | 14,73 | 25,88 |
| 39(1) | 410,58 | 408,08 | 1,58 | 1,25 |
| 40(1) | 403,61 | 396,68 | 5,03 | 5,30 |
| 41(1) | 348,20 | 343,31 | 0,19 | 0,17 |
| 42(1) | 337,48 | 334,05 | 5,18 | 4,94 |
| 43(1) | 237,97 | 237,69 | 0,02 | 0,02 |
| 44(1) | 167,10 | 166,32 | 0,05 | 0,04 |
| 45(1) | 158,88 | 156,96 | 3,24 | 3,15 |
| 46(1) | 111,99 | 111,99 | 0,03 | 0,03 |
| 47(1) | 86,29 | 86,29 | 2,33 | 2,33 |
| 48(1) | 6,46 | 6,46 | 3,09 | 3,09 |

**Phthalic acid overtones**

| Mode(n) | E(harm) | E(anharm) | I(anharm) |
| --- | --- | --- | --- |
| 1(3) | 11244,45 | 10438,89 | 0,056519 |
| 2(3) | 11243,05 | 10437,79 | 0,110929 |
| 3(3) | 9630,71 | 9109,19 | 0,001277 |
| 4(3) | 9621,02 | 9079,33 | 0,022992 |
| 5(3) | 9580,81 | 9079,15 | 0,014353 |
| 6(3) | 9539,21 | 8965,05 | 0,007645 |
| 1(2) | 7496,30 | 7125,63 | 0,000577 |
| 2(2) | 7495,37 | 6958,86 | 4,380673 |
| 3(2) | 6420,47 | 6147,31 | 0,002216 |
| 5(2) | 6387,21 | 6116,22 | 0,010078 |
| 4(2) | 6414,01 | 6070,10 | 0,074276 |
| 6(2) | 6359,47 | 5992,61 | 1,549737 |
| 7(3) | 5438,48 | 5311,51 | 0,019826 |
| 8(3) | 5380,47 | 5258,62 | 0,011407 |
| 9(3) | 4922,90 | 4809,78 | 0,006026 |
| 10(3) | 4871,94 | 4764,90 | 0,038396 |
| 11(3) | 4580,16 | 4448,71 | 0,002666 |
| 12(3) | 4426,45 | 4320,41 | 0,001130 |
| 13(3) | 4112,29 | 3988,28 | 0,000106 |
| 14(3) | 4102,04 | 3981,68 | 0,000262 |
| 15(3) | 4053,21 | 3952,04 | 0,000180 |
| 16(3) | 3902,49 | 3824,08 | 0,000591 |
| 7(2) | 3625,65 | 3552,86 | 0,911120 |
| 17(3) | 3656,87 | 3535,62 | 0,023558 |
| 19(3) | 3561,35 | 3532,91 | 0,000266 |
| 8(2) | 3586,98 | 3512,67 | 1,388540 |
| 18(3) | 3628,05 | 3489,63 | 0,013121 |
| 20(3) | 3474,34 | 3414,99 | 0,000577 |
| 21(3) | 3366,67 | 3266,90 | 0,000819 |
| 9(2) | 3281,93 | 3205,36 | 0,007705 |
| 10(2) | 3247,96 | 3175,75 | 1,825457 |
| 22(3) | 3200,79 | 3146,25 | 0,001813 |
| 23(3) | 3190,84 | 3136,52 | 0,077769 |
| 11(2) | 3053,44 | 2972,58 | 0,030734 |
| 24(3) | 3038,58 | 2946,17 | 0,000105 |
| 12(2) | 2950,97 | 2886,31 | 0,044544 |
| 25(3) | 2948,20 | 2875,60 | 0,000208 |
| 26(3) | 2725,09 | 2668,82 | 0,000001 |
| 13(2) | 2741,53 | 2662,89 | 0,367891 |
| 14(2) | 2734,70 | 2659,69 | 0,532370 |
| 15(2) | 2702,14 | 2635,99 | 0,218209 |
| 16(2) | 2601,66 | 2551,66 | 0,088967 |
| 28(3) | 2435,55 | 2406,48 | 0,000128 |
| 27(3) | 2435,94 | 2398,32 | 0,000022 |
| 29(3) | 2411,47 | 2365,38 | 0,001003 |
| 17(2) | 2437,91 | 2361,01 | 0,704320 |
| 19(2) | 2374,23 | 2352,01 | 0,355377 |
| 18(2) | 2418,70 | 2330,81 | 1,131574 |
| 20(2) | 2316,23 | 2276,97 | 0,038960 |
| 30(3) | 2257,03 | 2215,73 | 0,000054 |
| 31(3) | 2253,56 | 2207,30 | 0,001821 |
| 21(2) | 2244,44 | 2181,32 | 0,014455 |
| 32(3) | 2138,80 | 2100,06 | 0,000003 |
| 22(2) | 2133,86 | 2097,76 | 0,054852 |
| 23(2) | 2127,23 | 2092,39 | 0,011749 |
| 24(2) | 2025,72 | 1968,42 | 1,214037 |
| 25(2) | 1965,47 | 1919,06 | 0,346958 |
| 33(3) | 1912,70 | 1894,86 | 0,000062 |
| 34(3) | 1908,09 | 1888,96 | 0,000501 |
| 26(2) | 1816,72 | 1780,00 | 0,288295 |
| 36(3) | 1802,81 | 1726,69 | 0,000185 |
| 35(3) | 1816,12 | 1726,01 | 0,025882 |
| 37(3) | 1697,30 | 1672,96 | 0,057489 |
| 38(3) | 1643,63 | 1617,31 | 0,000115 |
| 28(2) | 1623,70 | 1604,68 | 0,050545 |
| 27(2) | 1623,96 | 1599,24 | 0,000393 |
| 29(2) | 1607,65 | 1577,43 | 0,771026 |
| 30(2) | 1504,69 | 1477,72 | 0,011743 |
| 31(2) | 1502,38 | 1471,90 | 0,353763 |
| 32(2) | 1425,87 | 1399,71 | 0,337937 |
| 33(2) | 1275,14 | 1262,86 | 0,037288 |
| 34(2) | 1272,06 | 1259,08 | 0,052503 |
| 39(3) | 1231,73 | 1223,98 | 0,016969 |
| 40(3) | 1210,83 | 1191,48 | 1,643087 |
| 36(2) | 1201,88 | 1164,70 | 1,233485 |
| 35(2) | 1210,75 | 1147,70 | 0,021902 |
| 37(2) | 1131,53 | 1114,62 | 2,851582 |
| 38(2) | 1095,75 | 1077,91 | 0,097286 |
| 41(3) | 1044,61 | 1029,39 | 0,000842 |
| 42(3) | 1012,44 | 1001,58 | 0,000917 |
| 39(2) | 821,16 | 816,07 | 0,020678 |
| 40(2) | 807,22 | 793,85 | 0,148976 |
| 43(3) | 713,90 | 715,54 | 0,000159 |
| 41(2) | 696,41 | 686,44 | 0,001546 |
| 42(2) | 674,96 | 667,92 | 0,060778 |
| 44(3) | 501,31 | 499,62 | 0,000016 |
| 43(2) | 475,93 | 476,20 | 0,000532 |
| 45(3) | 476,64 | 470,15 | 0,000026 |
| 44(2) | 334,21 | 332,86 | 0,006449 |
| 45(2) | 317,76 | 313,68 | 0,003045 |

**Mellitic acid fundamentals**

| Mode(n) | E(harm) | E(anharm) | I(harm) | I(anharm) |
| --- | --- | --- | --- | --- |
| 1(1) | 31,18 | 31,18 | 0,57 | 0,57 |
| 2(1) | 36,49 | 36,49 | 1,05 | 1,05 |
| 3(1) | 37,76 | 37,76 | 2,50 | 2,50 |
| 4(1) | 69,48 | 69,48 | 0,22 | 0,22 |
| 5(1) | 75,85 | 75,85 | 1,30 | 1,30 |
| 6(1) | 76,16 | 76,16 | 0,16 | 0,16 |
| 7(1) | 88,31 | 88,31 | 6,19 | 6,19 |
| 8(1) | 96,67 | 96,67 | 0,01 | 0,01 |
| 9(1) | 100,67 | 100,67 | 1,41 | 1,41 |
| 10(1) | 136,22 | 131,92 | 4,23 | 4,69 |
| 11(1) | 154,91 | 152,01 | 1,50 | 1,43 |
| 12(1) | 155,25 | 151,62 | 1,57 | 1,70 |
| 13(1) | 158,18 | 154,76 | 0,09 | 0,00 |
| 14(1) | 160,18 | 156,41 | 1,29 | 1,21 |
| 15(1) | 169,09 | 165,44 | 0,36 | 0,34 |
| 16(1) | 190,55 | 187,11 | 1,11 | 1,38 |
| 17(1) | 192,06 | 188,78 | 0,60 | 0,55 |
| 18(1) | 264,37 | 260,85 | 0,24 | 0,26 |
| 19(1) | 265,33 | 261,92 | 0,89 | 0,98 |
| 20(1) | 300,53 | 298,48 | 0,40 | 1,49 |
| 21(1) | 304,02 | 301,76 | 2,87 | 2,62 |
| 22(1) | 307,10 | 302,32 | 1,00 | 1,04 |
| 23(1) | 308,51 | 306,34 | 3,88 | 3,42 |
| 24(1) | 321,08 | 318,78 | 0,41 | 0,51 |
| 25(1) | 364,39 | 366,52 | 0,01 | 0,02 |
| 26(1) | 446,62 | 438,89 | 8,42 | 7,27 |
| 27(1) | 447,49 | 440,86 | 0,76 | 0,00 |
| 28(1) | 546,72 | 520,46 | 92,47 | 104,94 |
| 29(1) | 557,62 | 526,49 | 1,22 | 19,06 |
| 30(1) | 558,15 | 541,05 | 0,82 | 51,91 |
| 31(1) | 581,30 | 557,16 | 28,83 | 136,42 |
| 32(1) | 590,65 | 562,60 | 101,10 | 808,01 |
| 33(1) | 594,31 | 584,83 | 175,36 | 6,38 |
| 34(1) | 597,57 | 573,26 | 180,36 | 33,13 |
| 35(1) | 599,27 | 592,91 | 114,02 | 141,18 |
| 36(1) | 634,74 | 618,13 | 78,74 | 227,31 |
| 37(1) | 637,13 | 630,88 | 0,07 | 0,13 |
| 38(1) | 642,41 | 632,81 | 44,09 | 1,74 |
| 39(1) | 671,80 | 663,55 | 5,70 | 2,86 |
| 40(1) | 682,30 | 672,48 | 0,25 | 0,50 |
| 41(1) | 683,18 | 675,13 | 3,66 | 2,94 |
| 42(1) | 692,65 | 683,95 | 1,53 | 1,31 |
| 43(1) | 730,72 | 730,72 | 0,03 | 0,03 |
| 44(1) | 734,71 | 721,35 | 12,52 | 8,42 |
| 45(1) | 735,70 | 723,78 | 8,43 | 2,14 |
| 46(1) | 800,03 | 793,16 | 0,06 | 0,02 |
| 47(1) | 800,14 | 793,02 | 0,21 | 0,09 |
| 48(1) | 833,46 | 833,46 | 0,02 | 0,02 |
| 49(1) | 846,18 | 832,68 | 52,40 | 36,99 |
| 50(1) | 846,79 | 832,64 | 54,24 | 37,37 |
| 51(1) | 884,26 | 872,35 | 50,54 | 43,08 |
| 52(1) | 966,51 | 949,09 | 5,41 | 5,03 |
| 53(1) | 967,20 | 949,27 | 1,31 | 0,94 |
| 54(1) | 1120,61 | 1094,15 | 42,40 | 38,82 |
| 55(1) | 1156,08 | 1126,16 | 10,07 | 6,25 |
| 56(1) | 1167,62 | 1131,26 | 575,27 | 420,37 |
| 57(1) | 1173,35 | 1137,23 | 516,84 | 390,13 |
| 58(1) | 1183,28 | 1145,00 | 65,06 | 1,52 |
| 59(1) | 1187,25 | 1148,68 | 596,51 | 199,27 |
| 60(1) | 1239,46 | 1200,66 | 22,59 | 3,18 |
| 61(1) | 1258,32 | 1216,81 | 17,60 | 2,78 |
| 62(1) | 1322,56 | 1297,69 | 0,02 | 1,22 |
| 63(1) | 1330,04 | 1298,73 | 77,17 | 31,87 |
| 64(1) | 1333,28 | 1297,29 | 94,55 | 50,89 |
| 65(1) | 1361,14 | 1319,69 | 5,88 | 0,01 |
| 66(1) | 1361,45 | 1319,15 | 59,67 | 10,94 |
| 67(1) | 1394,73 | 1347,80 | 11,47 | 3,25 |
| 68(1) | 1419,41 | 1378,88 | 90,20 | 20,28 |
| 69(1) | 1461,07 | 1429,36 | 140,09 | 78,42 |
| 70(1) | 1463,74 | 1430,77 | 65,80 | 2,13 |
| 71(1) | 1606,27 | 1571,23 | 4,08 | 0,65 |
| 72(1) | 1606,93 | 1575,33 | 7,79 | 3,10 |
| 73(1) | 1812,84 | 1782,56 | 285,59 | 103,88 |
| 74(1) | 1812,86 | 1783,53 | 101,03 | 15,33 |
| 75(1) | 1815,05 | 1790,88 | 286,83 | 677,40 |
| 76(1) | 1816,27 | 1787,00 | 4,56 | 45,08 |
| 77(1) | 1817,04 | 1787,28 | 828,98 | 1124,44 |
| 78(1) | 1827,07 | 1795,36 | 302,82 | 211,21 |
| 79(1) | 3739,68 | 3549,49 | 15,29 | 109,07 |
| 80(1) | 3740,45 | 3557,62 | 12,11 | 199,32 |
| 81(1) | 3740,68 | 3530,02 | 12,92 | 76,85 |
| 82(1) | 3741,00 | 3557,34 | 300,76 | 63,17 |
| 83(1) | 3741,42 | 3554,23 | 314,87 | 178,08 |
| 84(1) | 3742,29 | 3576,40 | 8,97 | 7,04 |

**Mellitic acid overtones**

| Mode(n) | E(harm) | E(anharm) | I(anharm) |
| --- | --- | --- | --- |
| 10(2) | 272,43 | 263,17 | 0,000849 |
| 11(2) | 309,82 | 303,86 | 0,000003 |
| 12(2) | 310,49 | 303,50 | 0,155707 |
| 13(2) | 316,36 | 309,56 | 0,025162 |
| 14(2) | 320,36 | 312,68 | 0,048489 |
| 15(2) | 338,17 | 330,81 | 0,001436 |
| 16(2) | 381,09 | 374,55 | 0,004155 |
| 17(2) | 384,11 | 377,83 | 0,017278 |
| 18(2) | 528,74 | 521,64 | 0,000236 |
| 19(2) | 530,65 | 523,79 | 0,000195 |
| 20(2) | 601,07 | 597,41 | 15,601242 |
| 21(2) | 608,04 | 603,98 | 3,629703 |
| 22(2) | 614,19 | 604,46 | 0,113029 |
| 23(2) | 617,03 | 612,94 | 0,006739 |
| 24(2) | 642,16 | 637,63 | 0,027189 |
| 25(2) | 728,78 | 735,01 | 0,008721 |
| 26(2) | 893,24 | 877,50 | 0,035152 |
| 27(2) | 894,98 | 881,45 | 0,015843 |
| 28(2) | 1093,44 | 1057,89 | 0,154334 |
| 29(2) | 1115,24 | 1060,38 | 1,123649 |
| 30(2) | 1116,29 | 1067,53 | 0,000053 |
| 31(2) | 1162,61 | 1108,38 | 0,010471 |
| 32(2) | 1181,30 | 1117,86 | 0,653345 |
| 33(2) | 1188,61 | 1169,47 | 5,776336 |
| 34(2) | 1195,13 | 1138,86 | 0,586848 |
| 35(2) | 1198,54 | 1179,86 | 0,000601 |
| 36(2) | 1269,48 | 1235,68 | 0,089680 |
| 37(2) | 1274,25 | 1262,01 | 0,080210 |
| 38(2) | 1284,82 | 1264,79 | 0,338694 |
| 39(2) | 1343,59 | 1327,75 | 2,384439 |
| 40(2) | 1364,59 | 1346,26 | 0,837780 |
| 41(2) | 1366,35 | 1351,10 | 0,041597 |
| 42(2) | 1385,30 | 1367,89 | 0,156851 |
| 44(2) | 1469,42 | 1442,84 | 0,701190 |
| 45(2) | 1471,40 | 1445,41 | 0,121513 |
| 46(2) | 1600,06 | 1586,83 | 0,008216 |
| 47(2) | 1600,28 | 1586,63 | 0,303491 |
| 49(2) | 1692,35 | 1664,63 | 0,764412 |
| 50(2) | 1693,57 | 1664,64 | 0,113521 |
| 51(2) | 1768,52 | 1744,39 | 0,390328 |
| 52(2) | 1933,03 | 1897,65 | 0,182872 |
| 53(2) | 1934,40 | 1898,00 | 0,473461 |
| 54(2) | 2241,22 | 2187,48 | 0,353627 |
| 55(2) | 2312,15 | 2250,62 | 0,133033 |
| 56(2) | 2335,25 | 2259,86 | 0,124031 |
| 57(2) | 2346,70 | 2271,34 | 0,063049 |
| 58(2) | 2366,56 | 2285,63 | 0,259286 |
| 59(2) | 2374,50 | 2292,87 | 0,223256 |
| 60(2) | 2478,93 | 2399,99 | 0,000055 |
| 61(2) | 2516,65 | 2439,66 | 0,047617 |
| 62(2) | 2645,11 | 2596,05 | 0,063327 |
| 63(2) | 2660,07 | 2591,86 | 0,103609 |
| 64(2) | 2666,56 | 2600,48 | 0,000039 |
| 65(2) | 2722,29 | 2659,53 | 0,024822 |
| 66(2) | 2722,91 | 2661,22 | 0,004634 |
| 67(2) | 2789,45 | 2706,39 | 0,022234 |
| 68(2) | 2838,81 | 2755,66 | 0,026583 |
| 69(2) | 2922,15 | 2855,51 | 0,015656 |
| 70(2) | 2927,49 | 2856,20 | 0,019515 |
| 71(2) | 3212,54 | 3136,30 | 0,095142 |
| 72(2) | 3213,86 | 3144,50 | 0,003714 |
| 73(2) | 3625,67 | 3556,00 | 1,244500 |
| 74(2) | 3625,72 | 3552,16 | 0,006295 |
| 75(2) | 3630,09 | 3564,30 | 0,004631 |
| 76(2) | 3632,54 | 3567,45 | 0,570358 |
| 77(2) | 3634,09 | 3564,96 | 0,483935 |
| 78(2) | 3654,15 | 3586,69 | 0,775807 |
| 79(2) | 7479,35 | 7017,25 | 3,548743 |
| 80(2) | 7480,89 | 7049,56 | 3,298906 |
| 81(2) | 7481,37 | 7106,77 | 0,001779 |
| 82(2) | 7482,00 | 7111,16 | 0,000259 |
| 83(2) | 7482,84 | 6943,55 | 0,578742 |
| 84(2) | 7484,59 | 7109,47 | 0,000030 |

**Intermediate mellitic acid photoproduct fundamentals**

| Mode(n) | E(harm) | E (anharm) | I(harm) | I(anharm) |
| --- | --- | --- | --- | --- |
| 1(1) | 32,02 | 32,02 | 2,3414 | 2,3414 |
| 2(1) | 42,48 | 42,48 | 1,5845 | 1,5845 |
| 3(1) | 54,39 | 54,39 | 0,9355 | 0,9355 |
| 4(1) | 72,31 | 72,31 | 0,0003 | 0,0003 |
| 5(1) | 80,72 | 80,72 | 0,0013 | 0,0013 |
| 6(1) | 81,65 | 81,65 | 3,1369 | 3,1369 |
| 7(1) | 92,66 | 92,66 | 1,8432 | 1,8432 |
| 8(1) | 112,25 | 109,11 | 5,3865 | 5,4632 |
| 9(1) | 138,78 | 134,87 | 1,9641 | 1,7948 |
| 10(1) | 142,53 | 139,06 | 0,0142 | 0,0827 |
| 11(1) | 150,95 | 147,87 | 0,9605 | 1,3695 |
| 12(1) | 158,63 | 155,12 | 0,3786 | 0,4111 |
| 13(1) | 171,45 | 167,71 | 3,8233 | 4,0481 |
| 14(1) | 191,06 | 187,93 | 0,0161 | 0,0109 |
| 15(1) | 220,66 | 218,00 | 3,4893 | 3,4107 |
| 16(1) | 260,93 | 257,24 | 0,0355 | 0,0333 |
| 17(1) | 276,13 | 271,61 | 0,7564 | 0,8241 |
| 18(1) | 301,99 | 296,88 | 1,8350 | 2,0885 |
| 19(1) | 305,68 | 302,77 | 0,8175 | 0,6099 |
| 20(1) | 332,61 | 328,66 | 5,5456 | 5,7080 |
| 21(1) | 333,58 | 331,72 | 0,3799 | 0,3288 |
| 22(1) | 391,73 | 387,64 | 7,7273 | 7,5534 |
| 23(1) | 411,53 | 407,81 | 5,7896 | 3,1417 |
| 24(1) | 496,41 | 490,54 | 10,4158 | 10,6157 |
| 25(1) | 536,84 | 525,20 | 52,3220 | 67,6588 |
| 26(1) | 545,75 | 523,56 | 4,2053 | 16,7359 |
| 27(1) | 564,34 | 548,58 | 57,5200 | 59,6486 |
| 28(1) | 567,03 | 546,13 | 16,0947 | 17,7712 |
| 29(1) | 597,37 | 588,89 | 198,5509 | 174,6781 |
| 30(1) | 609,00 | 592,67 | 144,1720 | 110,3182 |
| 31(1) | 627,78 | 619,72 | 9,1038 | 27,6063 |
| 32(1) | 640,59 | 627,77 | 71,6513 | 89,1947 |
| 33(1) | 645,22 | 635,71 | 0,4940 | 0,2410 |
| 34(1) | 655,99 | 646,37 | 3,3177 | 1,6933 |
| 35(1) | 681,15 | 673,29 | 13,1678 | 11,3421 |
| 36(1) | 683,17 | 673,61 | 23,4508 | 21,2129 |
| 37(1) | 724,12 | 715,48 | 0,0321 | 4,5908 |
| 38(1) | 727,39 | 715,37 | 23,2332 | 13,6219 |
| 39(1) | 730,18 | 720,57 | 15,9833 | 16,3423 |
| 40(1) | 755,34 | 743,87 | 12,9173 | 9,9905 |
| 41(1) | 785,66 | 776,29 | 23,9392 | 19,2039 |
| 42(1) | 808,13 | 798,28 | 1,7583 | 1,2296 |
| 43(1) | 832,24 | 818,48 | 8,9408 | 4,0422 |
| 44(1) | 848,48 | 835,34 | 30,9943 | 28,3768 |
| 45(1) | 851,64 | 867,01 | 5,0303 | 0,8569 |
| 46(1) | 874,47 | 861,34 | 35,2348 | 26,7825 |
| 47(1) | 930,49 | 901,32 | 196,2067 | 165,1864 |
| 48(1) | 985,58 | 967,67 | 0,0019 | 0,1151 |
| 49(1) | 1007,38 | 986,58 | 11,2162 | 3,4058 |
| 50(1) | 1151,21 | 1121,53 | 26,4912 | 25,6899 |
| 51(1) | 1159,54 | 1130,29 | 7,3260 | 24,7814 |
| 52(1) | 1182,30 | 1143,95 | 287,3153 | 33,6045 |
| 53(1) | 1188,58 | 1148,87 | 997,3033 | 513,9179 |
| 54(1) | 1199,30 | 1166,17 | 108,4476 | 105,0619 |
| 55(1) | 1246,30 | 1210,88 | 1,8045 | 8,5608 |
| 56(1) | 1277,39 | 1228,29 | 104,5470 | 59,8020 |
| 57(1) | 1344,04 | 1317,43 | 16,5365 | 0,0447 |
| 58(1) | 1353,38 | 1317,11 | 203,2039 | 30,8753 |
| 59(1) | 1378,82 | 1350,90 | 0,1852 | 0,2152 |
| 60(1) | 1390,23 | 1358,29 | 1,7251 | 0,4667 |
| 61(1) | 1398,52 | 1367,31 | 36,5011 | 9,7905 |
| 62(1) | 1447,23 | 1412,78 | 47,4881 | 29,1038 |
| 63(1) | 1485,50 | 1447,24 | 79,5816 | 34,5356 |
| 64(1) | 1618,60 | 1587,67 | 0,1754 | 0,7436 |
| 65(1) | 1652,17 | 1618,57 | 12,3051 | 5,6102 |
| 66(1) | 1812,69 | 1780,57 | 257,8677 | 320,0764 |
| 67(1) | 1813,81 | 1782,22 | 2,4860 | 0,5911 |
| 68(1) | 1814,79 | 1777,42 | 670,1075 | 76,8362 |
| 69(1) | 1825,80 | 1791,93 | 308,0453 | 194,4674 |
| 70(1) | 1850,69 | 1817,87 | 569,8543 | 217,1293 |
| 71(1) | 1910,87 | 1899,16 | 213,8185 | 30,6346 |
| 72(1) | 3736,23 | 3552,01 | 182,6158 | 227,8309 |
| 73(1) | 3736,39 | 3552,26 | 32,7101 | 10,5780 |
| 74(1) | 3738,21 | 3556,44 | 152,8125 | 82,3252 |
| 75(1) | 3738,95 | 3557,03 | 127,5304 | 142,9096 |

**Intermediate mellitic acid photoproduct overtones**

| Mode(n) | E(harm) | E(anharm) | I(anharm) |
| --- | --- | --- | --- |
| 8(2) | 224,50 | 217,74 | 0,0029 |
| 9(2) | 277,55 | 269,74 | 0,0075 |
| 10(2) | 285,05 | 278,05 | 0,0057 |
| 11(2) | 301,90 | 295,69 | 0,0037 |
| 12(2) | 317,27 | 310,10 | 0,0034 |
| 13(2) | 342,91 | 334,70 | 0,0116 |
| 14(2) | 382,13 | 376,11 | 0,0000 |
| 15(2) | 441,33 | 436,10 | 0,0003 |
| 16(2) | 521,87 | 514,42 | 0,0002 |
| 17(2) | 552,26 | 543,26 | 0,0000 |
| 18(2) | 603,98 | 593,57 | 0,0047 |
| 19(2) | 611,36 | 605,62 | 0,0005 |
| 20(2) | 665,21 | 657,11 | 0,2086 |
| 21(2) | 667,15 | 663,16 | 0,0180 |
| 22(2) | 783,47 | 775,09 | 0,0411 |
| 23(2) | 823,05 | 815,45 | 0,1492 |
| 24(2) | 992,82 | 980,35 | 0,0975 |
| 25(2) | 1073,68 | 1049,71 | 0,0920 |
| 26(2) | 1091,49 | 1052,98 | 0,0241 |
| 28(2) | 1134,06 | 1088,53 | 0,3336 |
| 27(2) | 1128,68 | 1108,18 | 0,0479 |
| 29(2) | 1194,73 | 1161,48 | 12,1879 |
| 30(2) | 1218,00 | 1183,86 | 13,3826 |
| 31(2) | 1255,55 | 1239,22 | 1,6269 |
| 32(2) | 1281,18 | 1248,95 | 3,8733 |
| 33(2) | 1290,44 | 1270,93 | 4,3774 |
| 34(2) | 1311,97 | 1293,06 | 2,1779 |
| 35(2) | 1362,29 | 1346,61 | 0,0001 |
| 36(2) | 1366,35 | 1347,46 | 0,4041 |
| 38(2) | 1454,77 | 1430,37 | 0,3554 |
| 37(2) | 1448,25 | 1433,49 | 0,0608 |
| 39(2) | 1460,37 | 1441,08 | 0,0830 |
| 40(2) | 1510,69 | 1487,44 | 0,0448 |
| 41(2) | 1571,31 | 1552,32 | 0,0535 |
| 42(2) | 1616,26 | 1598,18 | 0,0002 |
| 43(2) | 1664,48 | 1636,40 | 1,1598 |
| 44(2) | 1696,96 | 1675,32 | 0,1023 |
| 46(2) | 1748,95 | 1722,12 | 0,6167 |
| 45(2) | 1703,28 | 1736,44 | 0,0002 |
| 47(2) | 1860,98 | 1792,51 | 4,4177 |
| 48(2) | 1971,16 | 1932,99 | 0,0676 |
| 49(2) | 2014,75 | 1973,44 | 1,9270 |
| 50(2) | 2302,42 | 2241,08 | 0,0749 |
| 51(2) | 2319,09 | 2258,10 | 0,0494 |
| 52(2) | 2364,60 | 2290,11 | 0,3016 |
| 53(2) | 2377,16 | 2294,44 | 0,2528 |
| 54(2) | 2398,61 | 2335,86 | 0,0006 |
| 55(2) | 2492,60 | 2426,35 | 0,0037 |
| 56(2) | 2554,77 | 2481,13 | 0,0081 |
| 57(2) | 2688,07 | 2628,33 | 0,0918 |
| 58(2) | 2706,76 | 2632,61 | 0,0080 |
| 59(2) | 2757,64 | 2692,63 | 0,3314 |
| 60(2) | 2780,46 | 2718,99 | 0,1570 |
| 61(2) | 2797,04 | 2732,36 | 0,0065 |
| 62(2) | 2894,45 | 2822,39 | 0,1424 |
| 63(2) | 2971,00 | 2892,20 | 0,0001 |
| 64(2) | 3237,20 | 3167,91 | 0,4461 |
| 65(2) | 3304,34 | 3228,36 | 0,1811 |
| 66(2) | 3625,38 | 3548,05 | 1,6644 |
| 68(2) | 3629,57 | 3548,49 | 0,8878 |
| 67(2) | 3627,61 | 3563,98 | 0,0030 |
| 69(2) | 3651,61 | 3578,83 | 0,6929 |
| 70(2) | 3701,37 | 3626,11 | 1,4160 |
| 71(2) | 3821,74 | 3756,55 | 1,1367 |
| 72(2) | 7472,46 | 6944,63 | 0,6030 |
| 75(2) | 7477,89 | 6970,45 | 5,1520 |
| 73(2) | 7472,78 | 7103,90 | 0,0035 |
| 74(2) | 7476,42 | 7114,12 | 0,0012 |

**Final mellitic acid photoproduct fundamentals**

| Mode(n) | E(harm) | E(anharm) | I(harm) | I(anharm) |
| --- | --- | --- | --- | --- |
| 1(1) | 52,87 | 52,87 | 1,16E-05 | 1,16E-05 |
| 2(1) | 52,90 | 52,90 | 0 | 0 |
| 3(1) | 62,63 | 62,63 | 0 | 0 |
| 4(1) | 106,90 | 106,90 | 17,48 | 17,48 |
| 5(1) | 153,55 | 147,73 | 1,80E-07 | 1,59E-04 |
| 6(1) | 153,59 | 148,10 | 5,97E-05 | 4,67E-04 |
| 7(1) | 161,92 | 157,60 | 5,23 | 5,09 |
| 8(1) | 162,58 | 158,67 | 1,09 | 1,23 |
| 9(1) | 162,64 | 158,19 | 1,09 | 1,19 |
| 10(1) | 279,00 | 270,56 | 0 | 7,35E-05 |
| 11(1) | 279,03 | 269,03 | 3,00E-08 | 4,32E-03 |
| 12(1) | 299,03 | 293,73 | 2,29 | 2,21 |
| 13(1) | 299,05 | 293,51 | 2,41 | 2,26 |
| 14(1) | 299,06 | 292,68 | 0,12 | 0,03 |
| 15(1) | 304,30 | 299,71 | 3,00E-06 | 8,02E-04 |
| 16(1) | 443,85 | 441,06 | 28,31 | 22,76 |
| 17(1) | 443,89 | 440,64 | 28,31 | 24,13 |
| 18(1) | 504,36 | 494,82 | 0 | 0,02 |
| 19(1) | 504,41 | 494,94 | 0 | 1,02E-04 |
| 20(1) | 523,89 | 516,09 | 9,90E-07 | 0,28 |
| 21(1) | 635,89 | 625,98 | 2,70E-04 | 0,02 |
| 22(1) | 636,66 | 629,46 | 0 | 0,01 |
| 23(1) | 636,80 | 626,78 | 1,22 | 1,26 |
| 24(1) | 636,81 | 626,71 | 1,21 | 1,43 |
| 25(1) | 686,30 | 675,20 | 1,89E-04 | 0,01 |
| 26(1) | 689,94 | 681,63 | 11,85 | 13,92 |
| 27(1) | 689,97 | 681,06 | 11,84 | 15,67 |
| 28(1) | 728,18 | 715,12 | 74,60 | 70,84 |
| 29(1) | 762,10 | 745,01 | 1,00E-08 | 0,08 |
| 30(1) | 762,13 | 745,14 | 5,70E-07 | 0,04 |
| 31(1) | 815,37 | 801,86 | 19,52 | 24,31 |
| 32(1) | 815,39 | 801,57 | 19,54 | 26,72 |
| 33(1) | 821,92 | 803,49 | 4,49E-06 | 4,63E-03 |
| 34(1) | 821,96 | 804,52 | 0 | 9,82E-04 |
| 35(1) | 827,83 | 818,36 | 1,56E-05 | 0,01 |
| 36(1) | 869,64 | 860,12 | 0 | 0,18 |
| 37(1) | 936,69 | 907,83 | 302,80 | 13,79 |
| 38(1) | 936,71 | 904,06 | 302,60 | 70,71 |
| 39(1) | 937,18 | 909,80 | 0,20 | 56,86 |
| 40(1) | 1036,50 | 1015,56 | 1,10 | 0,23 |
| 41(1) | 1036,52 | 1015,65 | 1,10 | 0,07 |
| 42(1) | 1151,30 | 1127,90 | 1,39E-05 | 0,31 |
| 43(1) | 1220,44 | 1193,54 | 490,54 | 351,25 |
| 44(1) | 1220,48 | 1193,70 | 490,55 | 356,38 |
| 45(1) | 1270,95 | 1252,89 | 3,21E-05 | 0,01 |
| 46(1) | 1294,74 | 1268,25 | 5,28E-06 | 1,09 |
| 47(1) | 1444,98 | 1413,60 | 0,86 | 0,14 |
| 48(1) | 1445,04 | 1413,80 | 0,86 | 0,08 |
| 49(1) | 1453,73 | 1425,62 | 2,22E-05 | 0,50 |
| 50(1) | 1664,66 | 1635,15 | 13,02 | 5,35 |
| 51(1) | 1664,67 | 1635,79 | 13,00 | 4,67 |
| 52(1) | 1853,71 | 1827,91 | 9,40E-06 | 0,01 |
| 53(1) | 1868,14 | 1831,42 | 600,83 | 199,64 |
| 54(1) | 1868,15 | 1830,97 | 600,59 | 235,39 |
| 55(1) | 1916,92 | 1896,62 | 354,56 | 263,44 |
| 56(1) | 1916,94 | 1897,05 | 354,77 | 186,17 |
| 57(1) | 1933,60 | 1908,87 | 3,30E-05 | 0,91 |

**Final mellitic acid photoproduct overtones**

| Mode(n) | E(harm) | E(anharm) | I(anharm) |
| --- | --- | --- | --- |
| 5(2) | 307,10 | 295,91 | 4,09E-05 |
| 6(2) | 307,17 | 296,59 | 0,02 |
| 7(2) | 323,84 | 315,32 | 1,32E-03 |
| 8(2) | 325,16 | 317,66 | 3,20E-03 |
| 9(2) | 325,28 | 316,63 | 2,85E-05 |
| 10(2) | 558,01 | 540,93 | 0,02 |
| 11(2) | 558,06 | 537,87 | 0,02 |
| 12(2) | 598,05 | 587,21 | 0,01 |
| 13(2) | 598,09 | 587,02 | 3,40E-03 |
| 14(2) | 598,12 | 585,60 | 6,98E-04 |
| 15(2) | 608,59 | 599,41 | 2,28E-06 |
| 16(2) | 887,70 | 877,15 | 0,49 |
| 17(2) | 887,77 | 876,68 | 0,49 |
| 18(2) | 1008,72 | 989,26 | 0,31 |
| 19(2) | 1008,81 | 989,59 | 0,31 |
| 20(2) | 1047,78 | 1031,89 | 2,00E-08 |
| 21(2) | 1271,79 | 1251,17 | 1,00E-08 |
| 22(2) | 1273,33 | 1259,10 | 7,00E-08 |
| 23(2) | 1273,59 | 1253,00 | 0,62 |
| 24(2) | 1273,62 | 1252,84 | 0,68 |
| 25(2) | 1372,59 | 1349,98 | 3,20E-07 |
| 26(2) | 1379,88 | 1363,26 | 0,05 |
| 27(2) | 1379,94 | 1362,11 | 0,06 |
| 28(2) | 1456,36 | 1429,84 | 7,00E-08 |
| 29(2) | 1524,20 | 1490,03 | 0,06 |
| 30(2) | 1524,27 | 1489,34 | 0,06 |
| 31(2) | 1630,74 | 1602,49 | 0,72 |
| 32(2) | 1630,77 | 1601,90 | 0,72 |
| 33(2) | 1643,85 | 1604,80 | 0,23 |
| 34(2) | 1643,91 | 1605,83 | 0,23 |
| 35(2) | 1655,65 | 1633,55 | 2,84E-06 |
| 36(2) | 1739,28 | 1723,57 | 0 |
| 37(2) | 1873,39 | 1808,55 | 79,52 |
| 38(2) | 1873,42 | 1802,65 | 42,63 |
| 39(2) | 1874,36 | 1813,12 | 27,48 |
| 40(2) | 2073,01 | 2030,35 | 0,44 |
| 41(2) | 2073,04 | 2030,50 | 0,43 |
| 42(2) | 2302,61 | 2254,54 | 4,00E-08 |
| 43(2) | 2440,88 | 2384,57 | 0,06 |
| 44(2) | 2440,95 | 2384,88 | 0,06 |
| 45(2) | 2541,91 | 2505,12 | 3,00E-08 |
| 46(2) | 2589,47 | 2532,70 | 1,00E-08 |
| 47(2) | 2889,96 | 2822,35 | 0,01 |
| 48(2) | 2890,08 | 2822,39 | 0,01 |
| 49(2) | 2907,46 | 2845,32 | 1,52E-06 |
| 50(2) | 3329,33 | 3261,42 | 0,05 |
| 51(2) | 3329,35 | 3262,86 | 0,05 |
| 52(2) | 3707,41 | 3637,30 | 0 |
| 53(2) | 3736,28 | 3657,39 | 0,21 |
| 54(2) | 3736,30 | 3657,33 | 0,21 |
| 55(2) | 3833,85 | 3788,42 | 0,02 |
| 56(2) | 3833,88 | 3788,45 | 0,02 |
| 57(2) | 3867,21 | 3811,23 | 3,50E-07 |

1. Arenas, J. F. & Marcos, J. I. Infrared and Raman spectra of phtalic, isophtalic and terephtalic acids. *Spectrochim Acta A* **36**, 1075–1081 (1980).

2. Colombo, L., Volovšek, V. & Lepostollec, M. Vibrational analysis and normal coordinate calculations of the o-phthalic acid molecule. *Journal of Raman Spectroscopy* **15**, 252–256 (1984).

3. Loring, J. S., Karlsson, M., Fawcett, W. R. & Casey, W. H. Infrared spectra of phthalic acid, the hydrogen phthalate ion, and the phthalate ion in aqueous solution. *Spectrochim Acta A Mol Biomol Spectrosc* **57**, 1635–1642 (2001).

4. Mishra, M. Fourier Transform Infrared Spectrophotometry (FTIR) Studies of Chromium Trioxide-phthalic Acid Complexes. *Chem Eng Trans* **5**, (2016).

5. Fornaro, T. *et al.* UV Irradiation and Near Infrared Characterization of Laboratory Mars Soil Analog Samples. *Frontiers in Astronomy and Space Sciences* **7**, (2020).

6. González-Sánchez, F. Infra-red spectra of the benzene carboxylic acids. *Spectrochimica Acta* **12**, 17–33 (1958).
